# Host control and the evolution of cooperation in host microbiomes

**DOI:** 10.1038/s41467-022-30971-8

**Published:** 2022-06-22

**Authors:** Connor Sharp, Kevin R. Foster

**Affiliations:** 1 Department of Zoology, University of Oxford, Oxford, UK; 2 Department of Biochemistry, University of Oxford, Oxford, UK

**Keywords:** Evolution, Microbiome, Gastrointestinal models

## Abstract

Humans, and many other species, are host to diverse symbionts. It is often suggested that the mutual benefits of host-microbe relationships can alone explain cooperative evolution. Here, we evaluate this hypothesis with evolutionary modelling. Our model predicts that mutual benefits are insufficient to drive cooperation in systems like the human microbiome, because of competition between symbionts. However, cooperation can emerge if hosts can exert control over symbionts, so long as there are constraints that limit symbiont counter evolution. We test our model with genomic data of two bacterial traits monitored by animal immune systems. In both cases, bacteria have evolved as predicted under host control, tending to lose flagella and maintain butyrate production when host-associated. Moreover, an analysis of bacteria that retain flagella supports the evolution of host control, via toll-like receptor 5, which limits symbiont counter evolution. Our work puts host control mechanisms, including the immune system, at the centre of microbiome evolution.

## Abstract (web summary)

Humans, and many other species, carry a large set of beneficial microbes. Here, the authors present new theory and data to argue that these vital relationships only work when hosts can control their microbiome and suppress wayward symbionts.

## Introduction

Humans, and many other multicellular organisms, are host to dense and diverse communities of microbial symbionts. These symbionts can provide a number of benefits, including nutrient provision, the promotion of immune system development, and protection against pathogens^1–5^. The benefits of carrying a microbiota are most discussed in mammals^6,7^, but are widely apparent, including in simple animals, like *Hydra*^8^, and plants^9^. These relationships also appear to benefit the microbes, through provision of nutrients and a relatively stable environment. The host-microbiota relationship, therefore, is typically characterised as one of cooperation and mutualism, where both sides receive considerable benefits^10^.

This characterisation has led to the conception of a host and its microbiota as a single evolutionary or organisational unit, sometimes known as the hologenome or holobiont hypothesis^11–17^. While this model may apply to some vertically-transmitted symbioses, such as intracellular bacteria of insects, many researchers have challenged the idea that host and symbionts are a unit, particularly for systems such as the human microbiome^18^. The key concern is the potential for strong evolutionary conflicts, both between the host and the microbiota and within the microbiota itself^19–22^. There remains a lack of clarity, therefore, on the evolutionary processes that drive cooperation between hosts and their microbiotas.

Evolutionary modelling allows one to dissect and explore the processes underlying the evolution of cooperation, and can both aid in the interpretation of existing data and generate new hypotheses for testing^23^. We decided, therefore, to use an evolutionary model of cooperation between species to explore host-microbiota systems. Based upon general theory developed for cooperation between species^24,25^, our model predicts little scope for cooperative evolution in systems like the mammalian microbiome that contain many strains and persist for multiple microbial generations^26^. However, our first model neglects a key piece of microbiome biology: the wide range of host mechanisms that may select against harmful strains and for beneficial ones^10,27–30^, including the innate and adaptive immune systems^31^. Introducing the potential for hosts to evolve such control mechanisms in the models, we find that they evolve and rescue cooperation, so long as symbionts are constrained from escaping the mechanism of control. We support our predictions with data from two key bacterial traits that influence the relationship with hosts and are monitored by the host immune system: possession of flagella and butyrate production.

## Results

### Theory: the barriers to cooperation within the microbiome

**Fig. 1:**
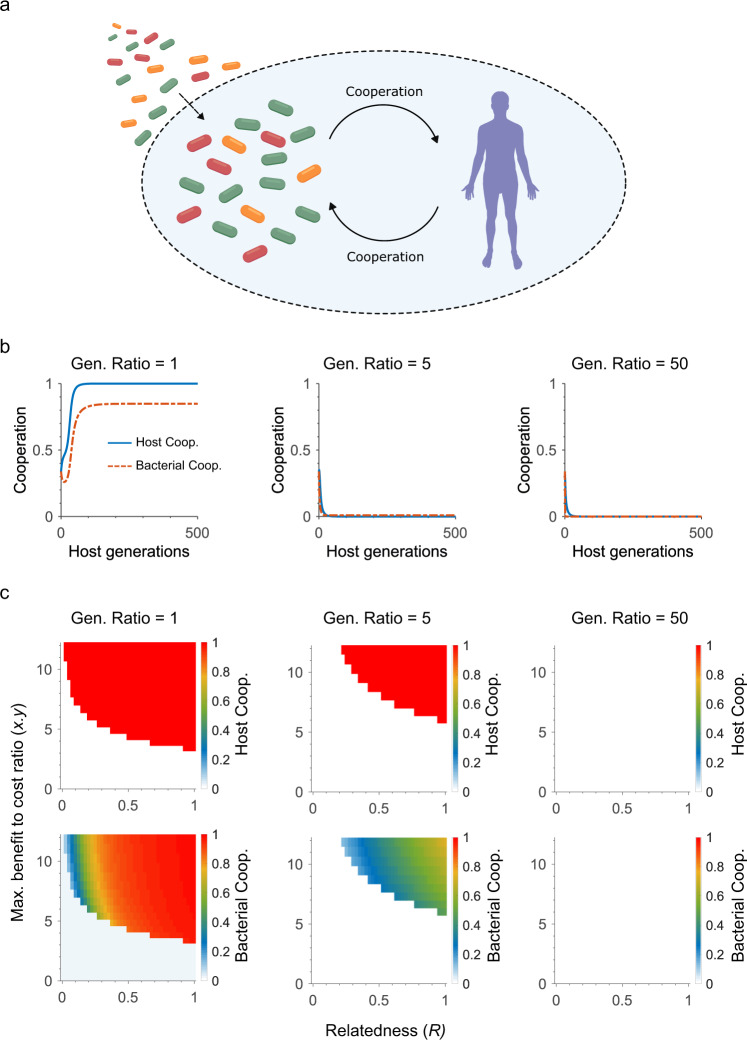
Cooperation breaks down in diverse and long-lived microbiomes. **a** Cartoon of the model: Both hosts and microbiota can invest in cooperation. Host can also invest in host control that preferentially benefits more cooperative symbionts. Microbes migrate into the system at rate *M* from a fixed environmental pool of largely uncooperative microbes between host generations, and at rate *m* each symbiont generation within host generations (Methods, Table 1). **b** Example dynamics from the model. Cooperation evolves when the benefits of cooperation are high, symbiont relatedness is high (i.e. within-species diversity is low) and the microbiome is short lived (the ratio of symbiont to host generations is 1). Increasing the number of symbiont generations within a single host generation (generation ratio) increases symbiont competition within the host and cooperation with the host collapses (unless stated, parameters are *x* = *y* = 2, *R* = 0.5, *f* = 0.02, *g* = 0.1, *m* = 1 × 10^−6^, *M* = 0.05). **c** Effect of relatedness and benefit to cost ratio of the evolution of cooperation. Cooperation is only stable at high relatedness, high benefit to cost ratio and low generation ratio. Increasing the generation ratio leads to the collapse of cooperation across a wide parameter space.

We focus on a host and its symbiotic microbes - where both sides of the relationship can evolve to invest in traits that provide a fitness benefit to the other (Fig. 1a, Methods, Table 1). For example, microbes could invest in production of a vitamin that benefits the host or simply evolve to be benign e.g. a strain that competes with pathogens and refrains itself from breaching the epithelial barrier, even though this restraint reduces its available nutrients. Hosts, meanwhile, might direct carbon towards the symbionts, such as the provision of glycosylated mucins.

**Table 1:** Parameters.

Parameter	Description
*a*	Investment in cooperation by the host.
*b*	Investment in cooperation by symbionts.
*B*	Expression of trait associated with cooperation by symbionts.
*c*	Investment in symbiont control by the host.
*f*	Cost of host control on symbionts: captures how much symbiont population size is reduced by control, which will indirectly affect the host by reducing its benefits.
*g*	Cost of control to host e.g. physiological cost of an immune system.
*H(a, c)*	Probability density of host individuals with cooperation level *a* and control level *c.*
*S(b)*	Probability density of symbiont genotypes with cooperation level *b.*
*M*	Rate of microbial immigration each host generation from a fixed environmental source population (proportion of resident population).
*m*	Rate of environmental microbe immigration each symbiont generation within the host.
*p*_*a*_	Effect of partner fidelity feedback on host individuals.
*p*_*b*_	Effect of partner fidelity feedback on symbionts.
*q*	Effect of host control on symbionts.
*W*	Fitness of focal individual.
*x*	Benefit to hosts of receiving symbiont cooperation.
*y*	Benefit to symbionts of receiving hosts cooperation.
*R*	Relatedness among symbionts (genetic similarity in the host within a species relative to the population mean).

Each host generation, microbes colonise new hosts from two sources. A proportion *M* comes from an environmental pool, which has not coevolved with the host and, therefore, has a low baseline level of cooperation. The rest of the microbes (1-*M*) come from the hosts of the previous generation, based upon their frequency there. If symbionts help their host, this will increase its fitness, and this effect can feedback as a benefit that increases the symbionts’ genotype in the next host generation (a between-host effect in the terminology of social evolution^23,32^). Intuitively, so long as the benefits are high and the costs are low, one might predict that cooperation will evolve under these circumstances. If the symbionts, for example, evolve some level of investment in the host, this can incentivise investment by the host in return, which in turn can favour further investment by the symbionts. However, there is a potential problem with this argument. The benefit to helping a host can be countered by competition between symbionts. This effect arises because genotypes that invest their energy in cooperation are expected to, all else being equal, have less energy for survival and reproduction than non-cooperative genotypes in the same host (a within-host effect).

### The effects of relatedness on cooperation

Many microbiomes are relatively open and diverse, which means a focal strain will experience competition from diverse microbial genotypes^10^. The question of how genetic diversity among social partners influences cooperation is central to evolutionary biology^23,33,34^, and captured by ‘relatedness’, *R* (Methods)^35^. Distinct from phylogenetic relatedness, this term in microbes captures the extent to which the genotype of a focal cell predicts the genotypes of all cells in the species under study^36^. In a simple case, with one strain, the focal cell genotype will predict all cell genotypes and *R* = 1. While, for ten randomly-selected strains, the genotype of any one cell will only predict one in ten of the cells’ genotypes and *R* = 0.1.

Why is this measure important? Consider when cooperation first emerges as a new symbiont genotype, such that the allele for cooperation is rare. When *R* = 1, if one cell cooperates with the host, all cells will as they are genetically identical, and all will share in the benefits, meaning that cooperation may readily evolve. By contrast, if *R* = 0.1, if one cell cooperates with the host, only one in ten cells will cooperate and yet all will again benefit from the cooperation. The effect is that the other 9/10 cells all get the benefit of cooperation without themselves paying the cost. The cooperative genotype, therefore, is likely to be outcompeted by these other strains. In this case, natural selection may favour symbionts that do not invest in cooperation, but receive any benefits from the cooperation of other symbionts in the microbiota. Over time, this can drive down the cooperation provided by the microbiota so far that the host no longer benefits from investing in the microbiota, and so cooperation is lost on both sides of the relationship.

We can see this effect as we decrease relatedness in the model—equivalent to increasing the number of different strains competing within the host—with a decrease in the region where cooperation is favoured (Fig. 1). Another key factor is the benefit to cost ratio: how much a recipient gains from cooperation relative to the costs of being cooperative. As relatedness is reduced, cooperation only evolves for a relatively high benefit to cost ratio (Fig. 1). Relatedness in the model captures the effects of competition between strains i.e. strains within the same niche in a host. However, a system like the human microbiome contains many such niches and many species that fill them. Here, a requirement for a high benefit to cost ratio may present a significant barrier to cooperation. With many species in a host, each symbiont strain is relatively rare and, all else being equal, less able to provide strong benefits for the host. This effect suggests that, in addition to the impact of low relatedness and competition within a given niche (Fig. 1), between-species diversity may also limit the evolution of cooperation in microbiomes.

### Chronic symbiont competition can be fatal for cooperation

A standard model of cooperation between species, therefore, suggests that systems like the human microbiome may have limited scope for cooperative evolution. However, missing from such models is the potential for there to be many symbiont generations per host generation. For example, one human generation can take ~30 y in contrast to symbiotic bacteria estimated to replicate on a timescale of hours^37^. This means that competition between strains is prolonged and chronic. Introducing this prolonged competition into the model (Methods) causes further problems for the evolution of cooperation (Fig. 1). Cooperating symbionts perform particularly poorly under these conditions, because their investment in the host makes them grow more slowly than symbionts that do not cooperate. The effect is to further decrease the likelihood of symbiont cooperation (i.e., at high ‘generation ratios’ in Fig. 1, Supplementary Fig 1). This, in turn, disincentivises the host from investing in the symbionts, which leads to a collapse of cooperation between host and microbiota.

This prediction is robust to changes in parameters and modelling assumptions. High generation ratios lead to the collapse of cooperation across broad parameter sweeps of both relatedness and the cost-to-benefit ratio of cooperation (Fig. 1c). The shape of the relationship between the investment in cooperation and its benefit can be important in some contexts^38,39^. We compared a range of functional forms relating symbiont cooperation to host benefit, and found consistently that cooperation collapses at high generation ratios (Supplementary Fig 1). Increasing symbiont immigration from the environment (*M*) to very high levels does generate cooperation. However, this only occurs because we assume a baseline level of cooperation in these immigrants, and this forcing effect on cooperation is again not robust to high generation ratios (Supplementary Fig 2).

Where does the human microbiome fit within these parameter sweeps? The available estimates for average symbiont relatedness is relatively high^40^ but, critically, the generation ratio is extremely high due to human life span being so long relative to that of microbes. These parameters again, therefore, lead to the prediction that cooperation will collapse due to competition within hosts (Supplementary Fig 3a).

### Host control can rescue cooperation in the microbiome

Our findings fit well with another recent model of host-microbiota evolution, which also concluded that the conditions for cooperation were very limited in systems like the mammalian microbiota^26^. However, we have so far overlooked the expectation that a host is under strong selection to promote symbiont cooperation^10,11,30^. Hosts can promote cooperation in a variety of ways, including selective feeding, influencing adhesion to the mucosa, and, of course, via the immune system^28–30^. Animal immune systems, for example, use toll-like receptors (TLRs) to detect conserved microbial features known as microbial associated molecular patterns (MAMPs), such as lipopolysaccharide and flagella. The presence of MAMPs can drive inflammation or other responses that targets and suppresses microbes^41^. Many of these mechanisms are of course already well known to counter specific pathogens^42–44^. Here, we are interested in their role more broadly in the evolution of a cooperative microbiota.

**Fig. 2:**
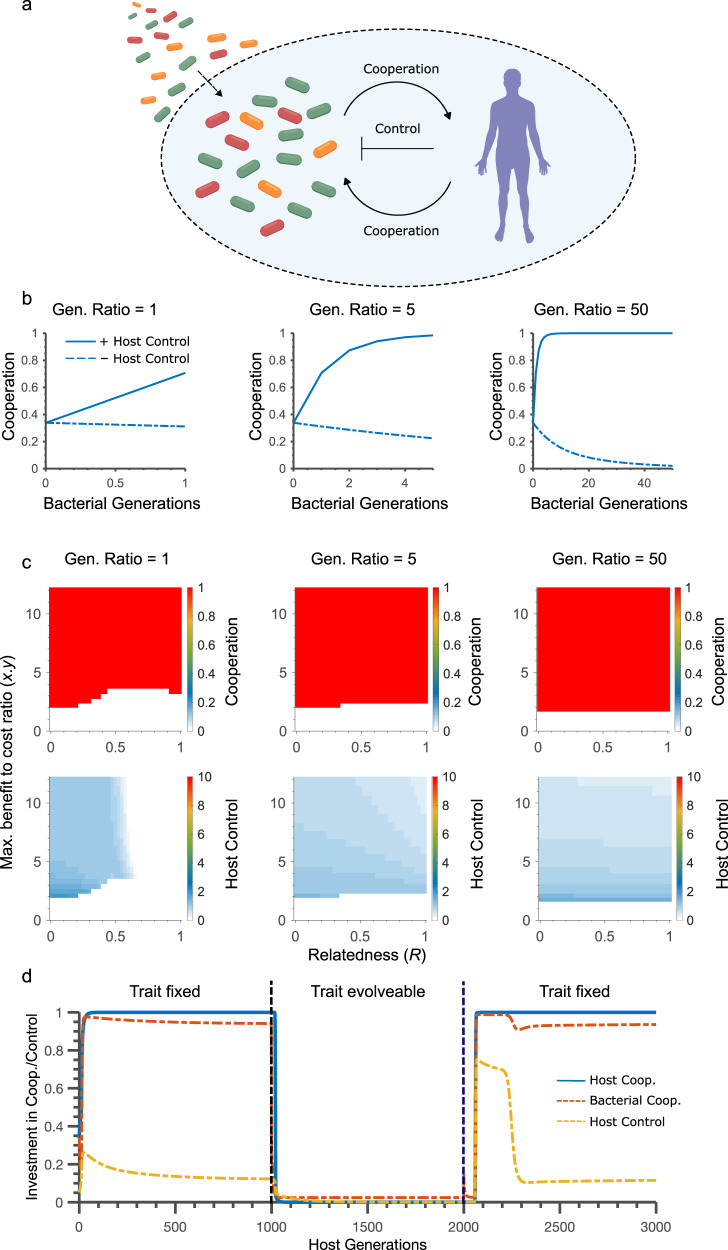
Host control stabilises the evolution of cooperation in the microbiome. **a** Schematic of the model: Both hosts and microbiota can invest in cooperation and, in addition, hosts can invest in control mechanisms that favour more cooperative symbionts over less cooperative ones. Hosts control also negatively effects all symbionts at cost (*f*) and hosts pay a direct cost for control (*g*). **b** Within-host evolution of symbiont cooperation (shown here for the first host generation, as an illustration). Increasing symbiont generations per host generation (generation ratio) promotes symbiont cooperation when there is host control, but hinders cooperation when there is not. **c**  Effect of relatedness and benefit to cost ratio on the evolution of cooperation. Cooperation evolves across broad parameter ranges with host control, where increasing the symbiont to host generation ratio only increases the range of conditions where cooperation is stable. The regions where cooperation evolves for hosts and symbiont overlap perfectly and so we show only a single plot for cooperation. **d** Cooperation collapses when symbionts can evolve cooperation independently of the trait that is the target of host control. Mutualism is stable while the trait and cooperation are fixed (original model) but when symbionts are allowed to evolve the trait-cooperation link, cooperation and control are quickly lost. Reinstating the relationship again renders host control effective and restores cooperation. Unless stated, parameters are: *x* = *y* = 2, *f* = 0.02, *g* = 0.1, *m* = 1 × 10^−6^, *M* = 0.05.

Our model predicts that allowing host control mechanisms to evolve will often rescue the evolution of cooperation (Fig. 2, Supplementary Fig 1)^25^. This prediction fits with a growing body of theory and data in social evolution supporting the importance of control (or ‘enforcement’) mechanisms for the evolution of cooperation, including a model of the plant microbiome^27,45^, When is host control most important for the evolution of cooperation? At low generation ratios, we find that control will only evolve under conditions where relatedness is relatively low. This result fits with classic evolutionary theory^46^ and occurs because host control is less effective and useful when relatedness is high. At higher generation ratios, the effects of relatedness are weakened by extended competition and evolution within the symbionts, and host control evolves across the whole range of relatedness (Fig. 2c).

At high generation ratios, host control also becomes more effective, because the selection imposed by hosts now acts across many symbiont generations and has a greater impact on genotype frequencies (Fig. 2b). Interestingly, this implies that the same property that can undermine cooperation in the microbiota of long-lived hosts (Fig. 1b, c) can help to rescue cooperation if there is host control (Fig. 2, Supplementary Fig 1). Consistent with this, when we again use parameters motivated by the human microbiome, our model predicts that host control can robustly rescue cooperation (Supplementary Fig 3b). We also provide parameter sweeps of the costs of host control (Supplementary Fig 4), the strength of host control (Supplementary Fig 5), and symbiont immigration rates from the environment (Supplementary Fig 6). As expected, higher costs of control result in hosts investing less in control at equilibrium. Nevertheless, across all parameter sweeps, the evolution of host control is widely predicted whenever there are a high number of symbiont generations per host generation. The same conclusion is reached when we consider the range of alternative relationships between symbiont cooperation and the benefit to the host (Supplementary Fig 1).

An exception to these conclusions occurs when there is no immigration of environmental symbionts, because here host control can collapse. This effect is well-known from previous models of enforcement^25,47,48^. Without immigration, host control drives all symbiont genotypes to be cooperative. This lack of symbiont variability means host control no longer has a benefit and is lost and with it, cooperation. In reality, there are many sources of symbiont variability, whether it is immigration or mutation, which means that host control is expected to be evolutionarily stable^25^. For example, in addition to general immigration of environmental genotypes (*M* in our model), an important source of such variability is the potential for pathogens. To account for this possibility, we developed an individual-based version of our model where we can follow a subset of immigrating genotypes that are especially costly for the host. As expected, including the potential for pathogens only increases natural selection for host control (Supplementary Fig 7b). This result underlines the potential for host control mechanisms, and indeed cooperation in the microbiome, to be shaped by pathogens that represent a particularly high risk to a host.

### Stable cooperation requires constraints on symbiont counter evolution

A final consideration is the potential for members of the microbiota to escape from mechanisms of host control. Specifically, natural selection is expected to favour symbionts that reduce their investment in cooperation, while keeping whatever trait the host targets to exert its control. We, therefore, asked what happens if symbiont evolution can alter the link between the trait under host control and their cooperation. Figure 2d shows the impacts of this change on evolutionary dynamics. When symbionts are constrained, cooperation and control both rapidly evolve. Indeed, host investment in control is greatest early on because this is when it is most needed to select cooperative symbionts. As symbiont cooperation increases, and symbiont variability decreases, host investment in control drops but to a stable level, which is set by the costs of control (above, Supplementary Fig 4).

This all changes when we remove the constraint on symbiont evolution. Now, symbionts rapidly evolve to maintain the trait under host control while reducing investment in cooperation. Host control becomes ineffective because it cannot select for the more cooperative symbionts, and is no longer favoured by natural selection leading to the collapse of cooperation (Fig. 2d). Another prediction of the model, therefore, is that cooperation rests upon the evolution of control mechanisms that cannot easily be escaped via counter evolution in the symbionts. This prediction is similar to the idea that the immune system needs to find conserved targets for pathogen recognition^44^, but here we are considering host control over the microbiota as a whole. As for our earlier results, parameter sweeps confirm that this prediction is robust to changes in relatedness and cost-to-benefit ratios (Supplementary Fig 8).

### Data: has host control shaped the evolution of animal microbiomes?

Our model predicts that host control mechanisms have been central to the evolution and maintenance of cooperation within diverse long-lived microbiomes, such as the human microbiome. The potential for host control is clear from the wide variety of mechanisms that can influence the microbiota, including the innate and adaptive immune systems of animals^10^. However, it is not known whether these mechanisms have been generally important for the evolution of host-associated microbiomes. A challenge for such a broad assessment is that the microbial traits associated with cooperation will typically differ among different host and symbiont species. We, therefore, sought a microbial trait that (i) is widely found and easily identified in genomic data (ii) influences whether symbionts benefit or harm the host and (iii) is subject to strong host control. These criteria led us to bacterial flagella.

### A test of the influence of host control using bacterial flagella

Many bacteria possess flagella, which are used to swim and move between microenvironments. Flagella can confer strong benefits to bacteria in a host. Swimming has been shown to help bacteria persist in the mammalian gut^49^ and, similarly, to escape peristalsis and ejection from the zebra fish gut^50^. For many pathogens, flagella are also essential for reaching the epithelial layer^51–53^. Due to this latter effect, flagella are important for cooperation and whether bacteria are likely to be beneficial to a host. Specifically, possession of flagella is often associated with harm to the host as a mechanism that allows bacteria to breach the epithelial barrier^50–54^ In *E. coli*, for example, only some strains appear to express flagella in the host, and these strains are associated with inflammation and disease^54^. Consistent with the importance for the host, the key structural component of bacterial flagella (flagellin) is amongst the most immunogenic of all microbial factors^55^, with a dedicated receptor in vertebrates (TLR5)^56^. Mice that lack this receptor have an increase in detectable flagellin in their microbiome^57^. Conversely, inducing the production of anti-flagellin IgA in mice decreases flagellin levels and limits the encroachment of the microbiota at the epithelial barrier^58^. Importantly, these experimental studies suggest that host control can limit flagellated bacteria and help in maintaining a cooperative relationship by preventing epithelial encroachment^56^. However, they leave open the question of how important these processes have been for the evolution of host microbiomes.

**Fig. 3:**
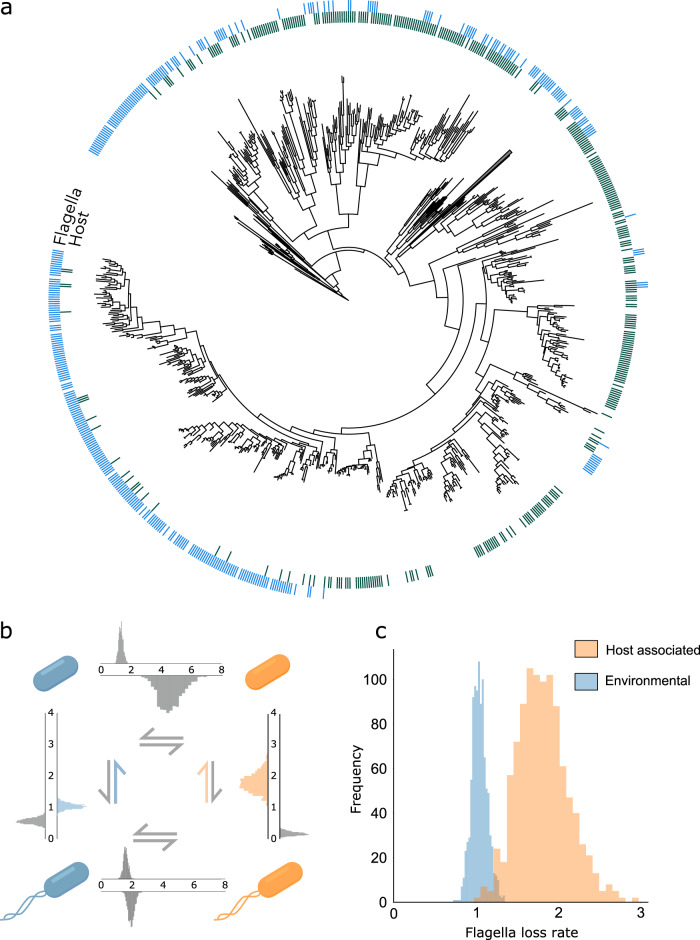
Host association and possession of flagella are negatively correlated, and host-associated bacteria have a higher rate of flagella loss. **a** 16 S phylogeny for strains in the PATRIC representative dataset. We only show Firmicutes here as an example because the full phylogeny is too large to show effectively. Host association was determined using metadata from the PATRIC and BacDive databases. Flagella status was determined by identified conserved motifs of flagellin genes. **b** Transitions between the four states in the data set, with and the posterior distributions of the transition rates calculated using Bayestraits^111^. **c** Posterior distribution of flagella loss rates for host-associated and environmental bacteria. Our model provides evidence for a significant difference in the rate of flagella loss between host-associated bacteria and environmental bacteria. Source data are provided as a Source Data file.

We therefore sought evidence—across animals—that host control mechanisms have served to suppress flagellated bacteria in spite of the documented benefits of swimming in the host^50–53^. We estimated both the frequency of flagellated species and the rate of flagella loss in environmental and host-associated bacteria using a database of 3833 sequenced bacterial strains (1262 host-associated and 2571 environmental)^59^ (see Materials and Methods) (Fig. 3a). Using the software BayesTraits, we assessed transitions between flagellated/non-flagellated and host/environmental bacteria, and fit the data to a simple model where the two traits are independent, and a complex model where rate of change in flagella status was dependant on host association status and vice-versa (Fig. 3b). Comparing the likelihood of both models, we can robustly reject the simple model in favour of a complex model where the two traits are dependant (Log Bayes Factor (LogBF) = 47.24). We tested for implicit biases in the dataset by performing 100 replicates with random label switching, which produced no significant results (LogBF = −42.73).

The supported model contains a number of transitions between states that could influence a link between flagella status and host status. To confirm that host association is driving the evolution of flagella loss, we examined the key transition rate from flagellated to non-flagellated bacteria. This analysis revealed that the data support a model where host association is predictive of flagella loss rate (LogBF > 2). Moreover, in line with the predicted effect of hosts control, flagella loss rates are higher in host-associated bacteria than in environmental strains (Fig. 3c).

### A second test of host control effects: butyrate production in the mammalian microbiota

**Fig. 4:**
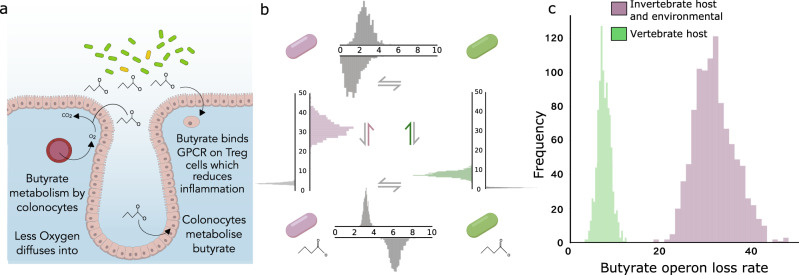
The pyruvate to butyrate operon is maintained over evolutionary time in host-associated bacteria. **a** Cartoon of butyrate biology: the short chain fatty acid is produced by members of the mammalian microbiome and is a key energy source for the host colonocytes. The anaerobic environment of the gut is favourable to butyrate producing bacteria and is reinforced by metabolism of butyrate by colonocytes, which lowers the oxygen potential in the gut. In addition, butyrate can reduce inflammation via effects on regulatory T cells by binding to G-protein couples receptors (GPCR)^60,61^, (**b**) Evolutionary loss rate of a pyruvate to butyrate operon based upon the genomes of the PATRIC database (Methods). **c** Posterior distribution for butyrate loss rates for symbionts associated with vertebrate hosts against environmental or invertebrate associated hosts. Source data are provided as a Source Data file.

The use of flagella by bacteria is associated with breaches of the epithelial barrier and inflammation^50–54^ and limiting flagella has the potential to improve the cooperativity of the microbiota^58^. However, in this case, ‘cooperation’ is the absence of a trait, rather than the presence of a trait that provides benefits to the host, which is a more typical example in the literature. We, therefore, sought a second independent test of the importance of host control, involving a beneficial microbial trait. In the mammalian gut, anaerobic bacteria produce short chain fatty acids, including butyrate, which is considered central to the host-microbiota relationship. Butyrate is a major source of nutrition for the colonic epithelium and is monitored by the immune system (Fig. 4a). Butyrate binds to G-protein coupled receptors in host cells, which influences the levels of regulatory T-cells and lowers intestinal inflammation^60,61^. In addition, butyrate is made by obligate anaerobes and so the maintenance of an anaerobic gut by a mammalian host^62^ is a second mechanism likely to favour butyrate production.

If host control is important, the prediction is that butyrate production will be better maintained (lost less often) in the mammalian microbiome relative to other microbiomes. To test this, we searched the same dataset as above for operons associated with butyrate production^63^, to study the loss rate of butyrate production across bacteria that live in different hosts and environments. Butyrate production may also be important for host physiology in vertebrates other than mammals^64^, and so we first compared loss rates in all vertebrate microbiotas (including mammals) versus all other microbiotas (Fig. 4). We also performed the more stringent test of mammal microbiotas versus all others. In both cases, the data support a model where host association and butyrate production are non-independent (LogBF = 58.37 for vertebrate analysis, LogBF = 45.77 for mammal analysis). Moreover, the loss rate is lower where we predict i.e. lower in vertebrate microbiotas than all others (LogBF = 36.17) (Fig. 4) and lower in mammalian microbiotas than all others (LogBF = 33.42).

### Evidence for escalation of host control and flagella loss in vertebrates

The data for both flagella and butyrate metabolism, therefore, are consistent with the prediction that host control—including immunological responses to bacterial traits—has influenced microbiome evolution and cooperation. Importantly, both tests could refute our hypothesis and yet both were consistent with our modelling predictions, and the published experimental work showing that the immune system can modulate bacterial traits in the microbiome^57,58^. However, both tests are also very broad, spanning a wide range of hosts (all animals) and symbionts (all bacteria). As a result, we cannot exclude the possibility that other factors are important in the patterns we observe. We, therefore, sought additional tests of our modelling predictions.

The flagella data set provided such an opportunity. Flagella are targeted by the invertebrate and vertebrate immune systems, but vertebrates show an elaboration of anti-flagella mechanisms. With vertebrates, there was the evolution of TLR5: a dedicated anti-flagellin receptor that mounts both innate and adaptive immune responses^56^, where the latter responses are absent in invertebrates that lack an adaptive immune system. The evolution of vertebrates is also associated with longer life and so a higher number of symbiont generations per host generation. Our model predicts that both of these effects—stronger host control and increased symbiont generations in a host—will promote flagella loss (Fig. 2, Supplementary Fig 5). We compared patterns of flagella loss evolution in vertebrate symbionts relative to invertebrates but this analysis lacked power using our original dataset (PATRIC^59^). While the trends looked encouraging, there were too few invertebrate species to resolve patterns. We were then fortunate that a new larger dataset was published: the Genomes of Earth’s Microbiomes, which is a collection of genomes assembled from metagenomic sequences from environmental samples and from a variety of hosts^65^.

We first used this new data set of 13757 taxa to confirm our original flagella analyses (shown in Fig. 3)^65^. This replicated the results of the PATRIC dataset in both the association of flagella and host-association traits (LogBF = 33.61) and even stronger evidence of a difference in the rate of flagella loss between host-associated and environmental bacteria (LogBF = 15.02). We next compared patterns in vertebrate vs invertebrate associated bacteria (3333 taxa in total). As predicted, we found a significantly higher flagella loss rate in vertebrate symbionts than invertebrate symbionts (LogBF = 6.14) (Supplementary Fig 9). This analysis, therefore, is again supportive of the predicted role of host control mechanisms in microbiome evolution.

### Counter evolution in the microbiome is constrained by TLR5 targeting

Whenever a host is able to drive bacteria to lose their flagella, this is likely to be an effective way to promote cooperation because it will limit their ability to reach host tissue^50–54^. However, there is the possibility that symbionts might evade the immune system without losing their flagella, via modifications that prevent the flagella being detected. Our models predict the need for constraints on such counter evolution in symbionts for host control, and cooperation, to be stable (Fig. 2d). We, therefore, explored the potential for counter evolution within the microbiome, as a final test of our modelling predictions. Here, we turned to the key mediator of flagella recognition in vertebrates, TLR5, which binds to flagellin, the main structural component of flagella. Consistent with ongoing host evolution, previous work found evidence that TLR5 is under positive natural selection^66–70^. For example, there is evidence that a core set of sites in TLR5 are under positive selection across all mammals^69^, with further residues that are positively selected within particular lineages or species^66,68,69^. Furthermore, differences in TLR5 are associated with host-specific phenotypes, with different host species responding to flagellins of different bacterial species with varying sensitivity^71–73^.

We looked for evidence that TLR5 evolution has driven comparable changes in the D1 domain of flagellin, which is the key region for TLR5 binding^74^. We studied the flagellin genes of six symbionts that are typically not pathogenic (*Butyrivibrio fibrisolvens, Citrobacter freundii, Clostridium butyricum, Enterobacter cloacae*, *Escherichia coli, Roseburia intestinalis*) and six major pathogens (*Burkholderia pseudomallei, Helicobacter pylori, Proteus mirabilis, Pseudomonas aeruginosa, S. typhimurium*, and *Vibrio cholerae*), all found in the human gastrointestinal tract. We included pathogens as we reasoned that evidence of counter evolution is most likely to be found there, and indeed might exclusively occur there, given the evolutionary pressures that hosts exert on pathogens^75,76^.

**Fig. 5:**
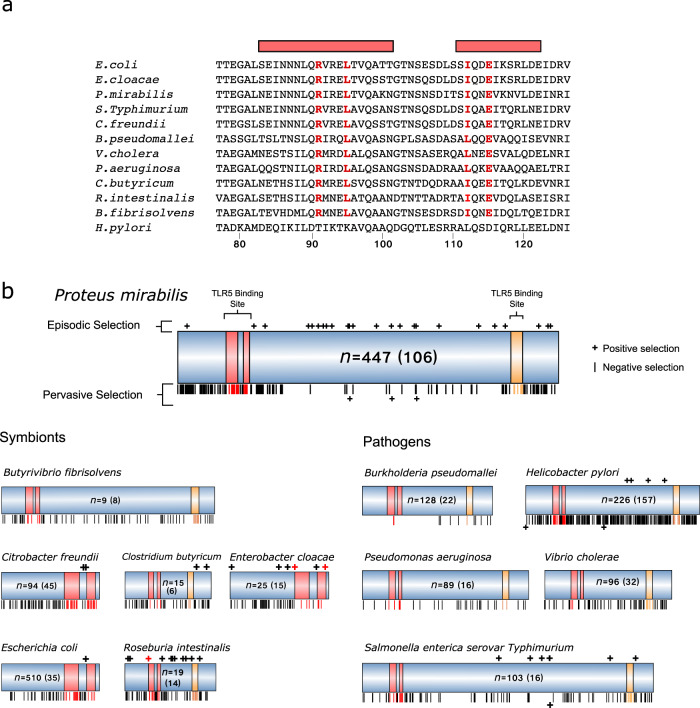
TLR5 targets a conserved region of bacterial flagellin within which we find little evidence of positive selection. **a** Alignment of the domain of flagellin which TLR5 recognises in symbionts and pathogens. Red bars indicate residues predicted to be in the interface between flagellin and TLR5^74^. Red residues have been identified as important for TLR5 binding by alanine scanning mutagenesis^74^. As a member of the ε-proteobacteria, *Helicobacter pylori* has managed to escape TLR5 recognition and maintain motility by a serious of compensatory mutations^74^. **b** Schematic of flagellin alignments for the 12 species tested. Numbers indicate the total number of sequences in the alignment (and the number of unique sequences). Red domains indicate the TLR5 binding region as shown in the above alignment, yellow domains are a second site that also interacts with TLR5 (a C-terminal region that also forms part of the D1 domain when the protein folds). Episodic positive selection was determined as any site with an LRT > 2 and *p* < 0.05 (calculated by MEME, and pervasive positive selection an ω > 1 and *p* < 0.05 calculated by FEL and are represented by ‘+’). Lines indicate pervasive negative selection at residues predicted by FEL to have a value of ω < 0.05. For *C. freundii*, *E. cloacae* and *E. coli* variable domains made aligning the full flagellin sequence inaccurate, therefore we focused only on the N-terminal D1 domain, which is the primary binding site for TLR5.

We examined flagellins in 1761 strains across our 12 species. In all 11 species which are expected to be recognised by TLR5, the four key residues shown to be important for TLR5 binding (by alanine-scanning mutagenesis^74^) were extremely highly conserved. Specifically, at these four residues, there was only one change from the consensus sequences (E115 to K115) in one *E. coli* strain out of a total of 1535 strains across the 11 species, which suggests little or no evolutionary escape from TLR5 recognition (Fig. 5a). Across species, one of the four residues (I112 in *E. coli*) is variable, but only between two similar hydrophobic amino acids (leucine and isoleucine) that are both known to allow TLR5 binding^74^. The exception that helps prove the rule is *H. pylori* flagellin which is not recognised by TLR5 and differs from the other species at three of the four key residues^77^.

Moreover, in contrast to host evolution in TLR5, we found few examples of positive selection in the TLR5 binding site for two measures of natural selection, across both the commensals and the pathogens (Fig. 5). The first measure (FEL)^78^ assesses pervasive selection i.e. natural selection that is consistent and relatively constant at a given site within the gene of interest. Here, the majority of sites identified were under strong pervasive negative (purifying) selection, which acts to limit evolutionary change. Moreover, all cases of positive selection were outside of the TLR5-binding D1 domain. The second measure (MEME) evaluates evidence for episodic site-specific selection where some alleles experience strong selection while others may not experience any^79^. This measure identified cases of positive selection across the species, which confirms there is statistical power to detect these sites. However, only three residues were in the D1 domain (two in *E. cloacae* and one in *R*. *intestinalis*) and then always on the very edge of the domain. In summary, we find that the key residues for TLR5 binding are highly conserved, and there is very limited evidence for positive selection in the D1 domain.

The data suggest distinct evolutionary patterns in the host and the microbiota. While host TLR5 appears free to evolve and tune its response to different bacterial flagella, the target of TLR5 in bacteria appears constrained. What drives this constraint? Part of it may be TLR5 itself, if this limits the sequences that bacteria use to those that are not highly immunogenic. However, a key cause is clearly structural. There is a highly conserved molecular interaction between the D1 and D0 domains of flagellin, which is critical to the polymerisation that builds the flagella. The importance of this region for flagella functioning was shown by detailed studies that mutated all residues in the D1 domain^80,81^. The great majority of residues are required for normal motility, suggesting that bacteria cannot easily change the D1 domain without affecting flagella functioning.

Our modelling predicts that for host control to be evolutionarily stable, it must target constrained bacterial traits that have limited potential for counter evolution, because otherwise bacteria are predicted to evolve to evade control (Fig. 2d). In support of this prediction, we find little evidence for functional evolutionary change in the region of flagellin that is targeted by TLR5. As discussed above for the case of *H. pylori*, the only flagellin where escape from TLR5 detection is documented is that of the α- and ε-Proteobacteria. These groups have a heavily altered TLR5 recognition region that does not illicit a TLR5 mediated immune response^77,82^. Importantly, to swim, these strains have also accumulated a series of compensatory mutations that maintain the flagella polymerisation and function^77^. This exception, therefore, is again consistent with there being a significant functional barrier to changes in the D1 region.

## Discussion

### Host control and microbiome evolution

It is common to assume that the potential for mutual benefits between host and microbiota is sufficient to explain their cooperation. By contrast, our modelling predicts that mutual benefits alone are not sufficient to maintain cooperation in diverse and long-lived microbiomes (Fig. 1). High diversity and the potential for evolution within a microbiome means that hosts need effective control mechanisms that favour more cooperative symbionts (Fig. 2). In support of the importance of host control for microbiome evolution, we found that host-associated bacteria are more likely to lose their flagella than environmental bacteria over evolutionary time (Fig. 3), which also fits with the large body of experimental evidence showing that the immune system selects against bacterial flagella. A competing explanation for the evolutionary pattern we have found is that host association, independently of the immune system, has selected against bacterial flagella. However, experimental work suggests the opposite: flagella help bacteria to compete and persist in the gut^49,50^. Moreover, we find in a second test case—butyrate production in the mammalian microbiome—that the evolutionary patterns again fit with the prediction from host control (Fig. 4). We also find that the elaboration of anti-flagella mechanisms and increase in host generation time in vertebrates is, as predicted, associated with an increase in flagella loss rate relative to invertebrate microbiomes. An interesting prospect for future work is a finer-grained evaluation of this last test that takes the generation times of diverse host species and relates this to the evolution of bacterial flagella.

Our model also predicts that symbionts must not be able to escape control mechanisms for them to be evolutionarily stable (Fig. 2d). This aligns with the general prediction from evolutionary biology that host control can generate pleiotropy at the loci for cooperation in the targeted species, because these loci now determine both the cooperative phenotype and any impacts of host control^27,83,84^. In the context of TLR5 evolution, the possession of flagella (reduced cooperation) becomes pleiotropically linked to increased targeting by the immune system. And, as predicted, the modern state appears to be one where counter evolution is limited because TLR5 targets a highly-constrained region of the flagella. Given its effectiveness, it is interesting that multiple animals have lost the TLR5 receptor^85,86^, including 5–10% people who have a the loss-of-function stop codon mutation TLR5^392STOP^^87^. However, while TLR5 loss in humans is linked positively to infection sensitivity, it is linked negatively with autoimmune disease, which may signify a cost from using the system to control the microbiota that can drive its loss^87–89^.

### Is there coevolution in the microbiome?

A key question in the study of the microbiome is the extent to which our beneficial microbes have coevolved with us^10,90^. Our models underline the fragility of cooperative coevolution to a diverse and long-lived microbiome. Specifically, the divergent interests of competing strains break down the coevolutionary feedbacks that can drive cooperation in mutualisms involving fewer partners. However, as for cooperation, we find that the introduction of host control mechanisms can rescue these coevolutionary processes. Is there evidence, therefore, that host control mechanisms have driven coevolution? Have there been successive stepwise evolutionary adaptation in mechanisms of host control and the targeted bacteria^91,92^. Our comparison of invertebrates and vertebrates is broadly supportive of a long-term coevolutionary dynamic, where hosts have progressively elaborated anti-flagella mechanisms and, on the other side, ever increasing numbers of symbiont species have lost their flagella (Supplementary Fig 9, above). However, as discussed above, there are other differences between invertebrates and vertebrates—notably host generation time—that may also explain the increase in flagella loss rate in vertebrate microbiomes relative to invertebrate ones.

Our analysis also raised the possibility of on-going coevolution between TLR5 and the D1 domain of bacterial flagellin. However, while TLR5 is commonly under positive selection, we found that the D1 domain of bacterial flagellin is highly conserved and almost exclusively under purifying selection (Fig. 5). This result suggests an absence of ongoing coevolution. However, unless TLR5 targeting evolved in one step, the modern state may reflect an ancient coevolutionary process where successive hosts explored different surveillance targets on bacteria until a suitably constrained target was found. Bacteria can also modulate the flagellin TLR5 interaction without altering the primary protein sequence^93^. One way to achieve this is to downregulate flagella expression^94^. The importance of this mechanism is supported by the data from *E. coli* discussed above, where only some strains express flagella in the host, and those that do are associated with inflammation^54^. Other mechanisms include glycosylation of flagellin^95,96^, sheathing flagella in lipids^97^, and the use of a secreted proteases that degrade free flagellin monomers^98,99^.

Interestingly, a new preprint (at the time of writing) suggests that the D0 domain of flagellin may, in some species, be modified in a way that limits TLR5 signalling by preventing its dimerization^100^. These ‘silent’ flagellins appear to be phylogenetically restricted, largely to *Lachnospiraceae* where the species that express them also carry flagellins which activate TLR5^101,102^. An interesting question for future research is whether the modifications to D0 interferes with normal functioning of the flagella, as seen with many changes to the D1 domain. Whatever the case, these observations raise the possibility for ongoing coevolutionary dynamics mediated by TLR5 recognition, which are not captured by sequence changes in the D1 domain of the flagellin gene. More broadly, our models predict that many interactions between host immunity and the microbiota are a potential source of coevolution in the microbiome e.g., the evolution of host antimicrobial peptides and symbiont resistance^103^.

### The ecosystem on a leash

Should hosts and their symbionts be considered holobionts that coevolve together and act as a single unit of natural selection^12^? Our work reinforces that systems like the mammalian microbiome—which are diverse and persist for many symbiont generations—do not act as a single unit of natural selection^10,18,26^. There is the potential for strong evolutionary conflicts between the host and the microbiota, and within the microbiota itself. However, our models also predict that, when host control is effective, these conflicts are reduced and cooperative coevolution can occur. Host control, therefore, has the potential to align interests in a way that brings a system closer to the notion of an integrated holobiont^17^. Importantly, this alignment is driven in the first instance by natural selection on the host to manipulate its microbiota, not because hosts and symbionts are a single unit of natural selection. Nevertheless, the results can be striking. In the bobtail squid and the luminescent bacterium *Vibrio fischeri*, for example, there is evidence for exquisite host control^104^ and a close functional integration of host and symbionts^27^. For systems like the mammalian microbiome, there is clearly lesser control and functional integration. Here, our work supports the idea of an ecosystem on a leash^10^, where the microbiome functions as a complex ecological system but natural selection for host control remains central to its evolution.

## Methods

### Model

There is a large body of theory on the evolution of cooperation, both within and between species, which has been at the forefront of evolutionary biology for over fifty years^23,33,34^. These models ask when will one individual invest in a cooperative trait, typically at a cost to itself, in order to benefit another individual of either the same or a different species. This approach has proved a powerful way to understand the conditions that favour cooperation and, by now, predictions have been supported by a large amount of empirical data from study systems as diverse as microbes, humans, birds, insects, and genomes^27,34^. However, while cooperation appears to be at the heart of the interaction between a host and its microbiota, these models remain little employed or discussed in the context of the human microbiome (see^26^ for a recent exception). We, therefore, decided to develop a model of microbiome cooperation, based upon earlier general models of cooperation between species^24,25^. Our framework employs methods that were developed by Frank and others^32^, but the core logic goes back to the classical papers of Hamilton and others that founded the modern field of sociobiology^33^.

We study a group of hosts A and symbionts B, where members of each can invest in cooperation that bring about benefits for their partner. While microbiomes often contain many microbial species, the model follows a focal species—or equivalently the members of one niche—and asks whether microbial strains will evolve to cooperate with the host. This focus allows us to predict evolutionary outcomes, because natural selection operates via competition within a population of a given species. However, as discussed in the main text, we can also use our model to broadly predict the effects of species diversity (the number of niches in a host). We also consider the possibility that hosts can invest in partner control *c*, which enables the host to preferentially benefit symbionts that are more cooperative. Finally, we assume that new microbial strains can migrate into the system from an environmental pool.

The fitnesses of hosts and symbionts are calculated from:

1 $${W}_{a}=\left(1-a\right)+x{p}_{a}\bar{b}\left(c\right)-{g}\left(\frac{c}{{c}_{max}}\right)$$

2 $${W}_{b}=\left(1-b\right)+y{p}_{b}{aq}\left(b,c\right)$$

where *W*_*a*_ is host fitness and *W*_*b*_ symbiont fitness, *a* and *b* represent the genetically-determined investment in cooperation by the host and symbiont respectively. The mean level of trait expression $\bar{b}$, is influenced by the effects of host control *c*. The parameters *x* and *y* determine the benefit to each species of receiving cooperation from the other. Hosts pay a direct cost to invest in control mechanism, *gc*, which could, for example, be the cost of carrying an immune system.

Host control influences symbionts based upon the host investment in control and the expression of a trait by symbionts *q*(*b,c*):

3 $$q\left(b,c\right)=\,\frac{{e}^{{bc}}}{{\int }_{0}^{1}{e}^{(1-R){bc}}S\left(b\right){{{{{\rm{d}}}}}}b}{e}^{-{fc}}$$

which calculates the effect of host control on a focal symbiont with cooperation level *b* within a host with control level investment *c*, where *S*(*b*) is the probability density of symbiont genotypes with cooperation level *b*. The exponential in the equation allows hosts to evolve a more effective control mechanism for higher values of *c*, but this comes a cost proportional to *c* (Eq. 1). *S*(*b*) defines the probability density of symbionts with trait expression level *b*. The denominator makes the impacts of control on a focal symbiont relative to the average trait expression across its competitors. We assume that the level of control is proportional to the genetic diversity among the symbionts, which is set by relatedness (1 - *R*), such that control does not discriminate within a clonal population of microbes (i.e., when *R* = 1). By relatedness here, we mean the quantity from social evolution theory, which is distinct from phylogenetic relatedness between strains or species. This quantity captures genetic diversity within a set of strains in an ecological niche: it is the probability above the population average, that two cells are genetically identical at the locus that drives cooperation^36^. When *R* = 1, there is a single strain, while if there were ten equally-abundant competing strains, *R* = 0.1. We also allow for the possibility that the act of host control has a negative effect on all symbionts, which is expected whenever hosts use control mechanisms such as antimicrobial peptides that reduce symbiont population sizes. We weight this effect with parameter *f*, which also leads to a negative feedback on host fitness.

Host control increases the frequency of symbionts which express cooperation at a higher level relative to others in the population. Mean trait expression after control is:

4 $$\bar{b}\left(c\right)=\,\frac{{\int }_{0}^{1}q\left(b,c\right)S\left(b\right)b{{{{{\rm{d}}}}}}b}{{\int }_{0}^{1}q\left(b,c\right)S\left(b\right){{{{{\rm{d}}}}}}b}$$

Where again *q(b, c)* is the effect of host control *c* on symbiont genotypes with a level of cooperation *b* and *S(b)* is the probability density of symbiont genotypes with cooperation level *b*.

In a mutualistic relationship, providing aid to one species can increase the ability of that species to return aid, such as when a host increases the population size of its microbial symbionts by feeding them. Such effects are often known as partner fidelity feedback^24^, which captures whether two partners stay together for long enough for any feedback benefits to return to a cooperative individual. In practice, these feedbacks may still occur with relatively short associations between partners. We define the potential for these effects as follows:

5 $${p}_{a}={ay}$$

6 $${p}_{b}\left(c\right)={Rbx}+(1-R)\,\bar{b}\left(c\right)x$$

where *p*_*a*_ is the partner fidelity feedback effect for the host, which is equal to the benefits that the symbionts receive from cooperation. The feedback benefit received by the symbionts from the host depends upon i) their relatedness (*R*), which here determines the relative importance of host control for the cooperation received by the host, and ii) the strength of benefits to a host from microbiota cooperation (*x*).

We assume a large population of hosts and symbionts where all host genotypes interact with all symbiont genotypes each generation, such that each symbiont genotype that exists in the population at a given time will experiences every level of host cooperation and control that is present. Using these assumptions, we have a final equation for symbiont fitness:

7 $${W}_{b}=\left(1-b\right)+y{\int }_{0}^{1}{\int }_{0}^{{c}_{{\max }}}H\left(a,c\right)q\left(b,c\right){p}_{b}\left(c\right)a{{{{{\rm{d}}}}}}c{{{{{\rm{d}}}}}}a$$

Where *W*_*b*_ is symbiont fitness, *b* the level of symbiont cooperation and *H(a,c)* is the probability density of host individuals with cooperation level *a* and control level *c*.

We also extend the model to capture the effects of there being multiple microbial generations for each host generation. Here, we use the above equation to capture symbiont fitness between host generations, while within host generations, we assume that symbiont fitness is defined by:

8 $${W}_{b}=\left(1-b\right)+{\int }_{0}^{{c}_{{\max }}}q\left(b,c\right){{{{{\rm{d}}}}}}c$$

where the symbionts compete based upon their relative growth rates i.e., pure local competition in the terminology of social evolution. Here, we assume that there is always genetic variability within hosts upon which natural selection can act. This assumption is made because, even if a single strain colonises a host (*R* = 1), mutation and strain immigration are expected to ensure there are additional genotypes upon which selection can act across multiple symbiont generations. Between host generations, symbionts also compete in their ability to disperse and colonise new hosts as before.

We use simulations to study the model’s behaviour where host populations are modelled as a 11 × 11 matrix which defines the proportion of the population with trait values *H(a, c)*, with *a*(0, 1) and *c*(0,*c*_*max*_). Microbes are similarly modelled as a 11 × 1 vector representing the proportion of the population with cooperation value *b*(0, 1). The evolution of cooperation between species is dependent upon initial conditions^24,25^. In particular, if there is too little cooperation in one species, the other species will not benefit from investing in cooperation, and so cooperation cannot get off of the ground. Only if a finite amount of cooperation is present in both species at the initial conditions, therefore, does cooperation have a chance^24,25^. Accordingly, we start our models with truncated normal distributions for cooperation with standard deviation of 0.5 for cooperation in both species, which gives a small quantity of cooperation in both partners from the beginning of the model. For control, we again assume that there is some pre-existing variation in the trait as otherwise cooperation is again prone to collapse before control can take effect (standard deviation = 1).

The benefits that symbionts provide to a host can vary greatly in type and quality between symbionts and systems. In our main model, we assume a simple linear relationship between cooperation in the symbionts and the benefits to the host. In reality, this relationship will often be non-linear. We therefore evaluated three different relationships, one weighted to assume that the benefits to the host saturate with increasing levels of investment by the symbionts  (Diminishing returns, ${benefit\; to\; host}=1-\frac{1-b}{1-6b}$), another which assumes that the benefits accelerate with increasing investment by the symbionts (Accelerating returns, ${benefit\; to\; host}=\frac{b}{b+0.3(1-b)}$), and finally we test a sigmoidal curve where significant benefits of the trait are only felt by the host above a certain threshold of expression in the symbiont (Sigmoidal returns, ${benefit\; to\; host}=\frac{1}{{(1+\frac{b}{1+b})}^{-6}}$).

### Modelling the effects of microbial escape from host control

Our models predict that host control is instrumental in the evolution of cooperation between hosts and their microbiota. However, this prediction comes from models that did not consider the potential for members of the microbiota to escape from the mechanism of host control. Natural selection is expected to favour symbionts that reduce their investment in cooperation, while maintaining the trait that the host targets for control. To account for this possibility, we extended our main model to allow for symbiont evolution in the trait that is the target of the host control mechanism. Specifically, we extended the model to allow evolution in the strength of the link between cooperation by microbes and their expression of a trait recognised by host control. To do this, we added an additional parameter (γ) which defines the relationship between cooperation (*b*) and trait expression (*B*). When *γ* = 1, the model behaves as before with a strict linear relationship and when γ = 0 the link is broken, and the hosts select against a trait which is no longer linked to cooperation. Microbial traits are then a 11 × 11 matrix with *B*(0,1) and *γ*(0,1) where

9 $$b=\gamma B$$

### Modelling the effect of pathogens on the evolution of host control

Some members of the microbiota have the potential to be especially costly for a host. These are the specialist pathogens, such as *Salmonella enterica* in the mammalian microbiome, which competes for the same niche as non-pathogenic *E. coli* strains. And within *E. coli*, there are both pathogenic and non-pathogenic strains. There is a large literature on host-pathogen evolution^75,76^, and we do not consider it in detail here. However, it is interesting to ask how the presence of pathogens might influence the evolution of the microbiome more generally. To capture this effect, we developed an individual-based version of our model. Here, we defined 10^4^ hosts with values of cooperation and control. Each host can carry 1000 individual microbes. We then defined 10^7^ microbes to occupy the hosts. Initial populations are defined as a normal distributions *N*(0, 0.5) truncated between 0 and 1 or *c*_*max*_ for host control. We use the previous fitness equations to define the frequencies of different trait values in the next generation. Each generation of hosts is occupied by a random subsample of microbes from the previous generation. We simulated a small number of microbes which fully express the trait, do not cooperate with the hosts (*b* = 0) and can drastically reduce fitness in a manner different from simply a lack of cooperation. We defined a pathogenicity factor *p*_*f*_, which captures the harm to a host from pathogens:

10 $${p}_{f}=\,{e}^{{vp}(\frac{c}{{c}_{{{\max }}}})}\,\times \,{e}^{-{vp}}$$

which is determined by the level of host investment in control (*c*), the proportion of pathogens in the host (*p*), and the virulence of pathogens (*v)* (where the exponent allows us to capture a high cost for the presence of even a small number of pathogens within a host). This new model enables us to capture an influx of rare but costly pathogenic microbes in addition to the symbionts, which is not possible to do explicitly with the original model. We assume that the pathogens are subject to host control in the same way as non-cooperative symbionts, but if the pathogens are able to persist they cause a much more severe decrease in host fitness than non-cooperative symbionts.

### Identifying flagellins

Presence of flagella was determined by identifying proteins which contained both the conserved Flagellin_N (PF00669.15) and Flagellin_C (PF00700.16) domains. These domains are conserved in both the flagellin monomers and other structural proteins such as the flagella hook protein.

### Positive selection of flagellin proteins

Species data was downloaded from the PATRIC database^59^. Blastp was used to identify the major flagellin in each genome. Sequences were aligned using PRANK with the codon aware alignment flag^105^. Alignments were analysed for episodic positive selection using MEME^79^ and pervasive selection using FEL^78^ on the Datamonkey server^106^. For MEME, positive selection was considered as any site with a Likelihood ratio test >2 supported by a *p* < 0.05. For Fel, negative selection was considered any site with ω < 0.05, positive selection ω > 1 and supported with a *p* < 0.05.

### Tree building

Genomes from the PATRIC reference database with an annotated 16S gene were used for alignment with Clustal Omega^107^. Phylogenetic trees were inferred using FastTree V2 with a general time reversible model and a Gamma distribution^108^. FastTree was selected over other software such as RAxML as it has been shown to have better performance in terms of both accuracy and computational efficacy on large 16 S datasets^109^.

### Ontologies

Host/environmental association of bacteria was determined using annotations from the PATRIC and BacDive database^59,110^. Both databases provide in depth descriptions of the original point of isolation on a strain specific level which we use as a proxy for bacterial niche. If niche annotations were conflicting in the PATRIC database—recorded as both isolated from an animal host and environmental source—we classified the strain was classed as environmental as it is unlikely to be a host specialist.

### Bayestraits analysis

Two datasets were used for the analysis of flagella loss, Pathosystems Resource Integration Center (PATRIC^59^) and Genomes of earths microbiomes (GEM^65^). PATRIC is a large dataset and series of analysis tools for bacterial genomes, and GEM contains metagenomes assembled from environments around the globe.

Bayestraits V3 was used to test for rates of loss for flagellin between host-associated and environmental bacteria^111^. Host association status was described as a binary trait (0: Environmental, 1: Host-associated) and flagellin presence/absence as a separate binary trait. Association between the two binary traits was determined by running two models. The first ‘independent’ model predicts the likelihood under the assumption that the traits evolved independently e.g., the rate of flagellin loss/gain is independent of host association. The second ‘dependant’ model assumes that the traits are dependent on each other. To test if the rates of flagella loss are different between host and environmental bacteria, we ran a third model where the rate of flagella loss was assumed to be equal in host and environmental bacteria, and this was compared to the dependent model. Significance between the models was determined by calculating the Log Bayes Factor (LogBF), a comparison of the marginal likelihood between different models, used to estimate the strength of the evidence favouring the hypothesis over null hypothesis. LogBF > 2 can be interpreted as significant evidence, LogBF > 5 is strong evidence and LogBF > 10 is very strong evidence favouring the complex model.

The BayesTraits Stepping stone sampler was used to estimate the marginal likelihood with 500 stones sampled over 20,000 iterations. To limit prior bias, hyperpriors were used for all analysis, drawing prior distributions for all parameters from exponential distributions with mean between 0 and 10. As our dataset is heavily skewed towards vertebrates, we tested for an implicit bias by performing 100 replicates with random label switching which produced no significant results and mean Log Bayes Factor of −42.73.

The GEM dataset was further split into vertebrate, invertebrate hosts and environmental microbiomes^65^. In a few cases, multiple niches or flagella statuses were found within a single OTU, and these taxa were removed from the analysis.

There is a concern that phylogenetic analyses can give spurious results if one of the traits has only a singular evolutionary transition^112^. To be confident that a single, or small number, of transition/s are not dictating our findings, we estimated the number of transitions in our traits across our trees using the R package phylotools and Simmap^113^. This analysis gave estimates of 441 transitions in flagella status and 1160 transitions in host status in the PATRIC data set, 1670 transitions in flagella status and 770 transitions in host status across the GEM dataset, and 1471 transitions in butyrate status and 1218 transitions in mammalian host status in the PATRIC data set. The patterns we observe, therefore, do not involve a small number of transitions in any of the traits concerned.

### Identifying butyrate systems

Bacteria which possess the genes responsible for fermenting pyruvate to produce butyrate were identified using Macsyfinder and PFAM (Table 2)^114^. To classify as a pyruvate fermenting pathway, we set the condition that bacteria must contain all 5 domains with an allowed intergenic distance gap of 5 genes following^63^.

**Table 2:** Pfam profiles used to identify the pathway for pyruvate fermentation.

Gene	Pfam profile
Thiolase	PF02803.13
3HCDH	PF00725.17
ECH	PF00378.15
Acyl CoA	PF02771.11
ETF	PF01012.16

### Reporting summary

Further information on research design is available in the Nature Research Reporting Summary linked to this article.

## Supplementary information

Supplementary Information

Reporting Summary

## Data Availability

Genomic data used in this study was accessed from publicly available datasets: Pathosystems Resource Integration Center (PATRIC) reference genomes (https://www.patricbrc.org) and Genomes of earths microbiomes (available at https://img.jgi.doe.gov/ and https://portal.nersc.gov/GEM). Additional metadata was accessed from the publicly available dataset BacDive (https://bacdive.dsmz.de). Source data are provided with this paper.

## Source data

Source Data

## Code availability

Evolutionary modelling was performed using scripts found at https://github.com/Connor-Sharp/CoevolutionModel^115^.
