# Supplementary material for: Host control and the evolution of cooperation in host microbiomes
Source: Nat Commun. 2022 Jun 22;13:3567. doi: 10.1038/s41467-022-30971-8 (PMC9218092; doi:10.1038/s41467-022-30971-8)
Supplement: Supplementary file 1 — Supplementary Information [file 41467_2022_30971_MOESM1_ESM.pdf]

Supplementary Figures

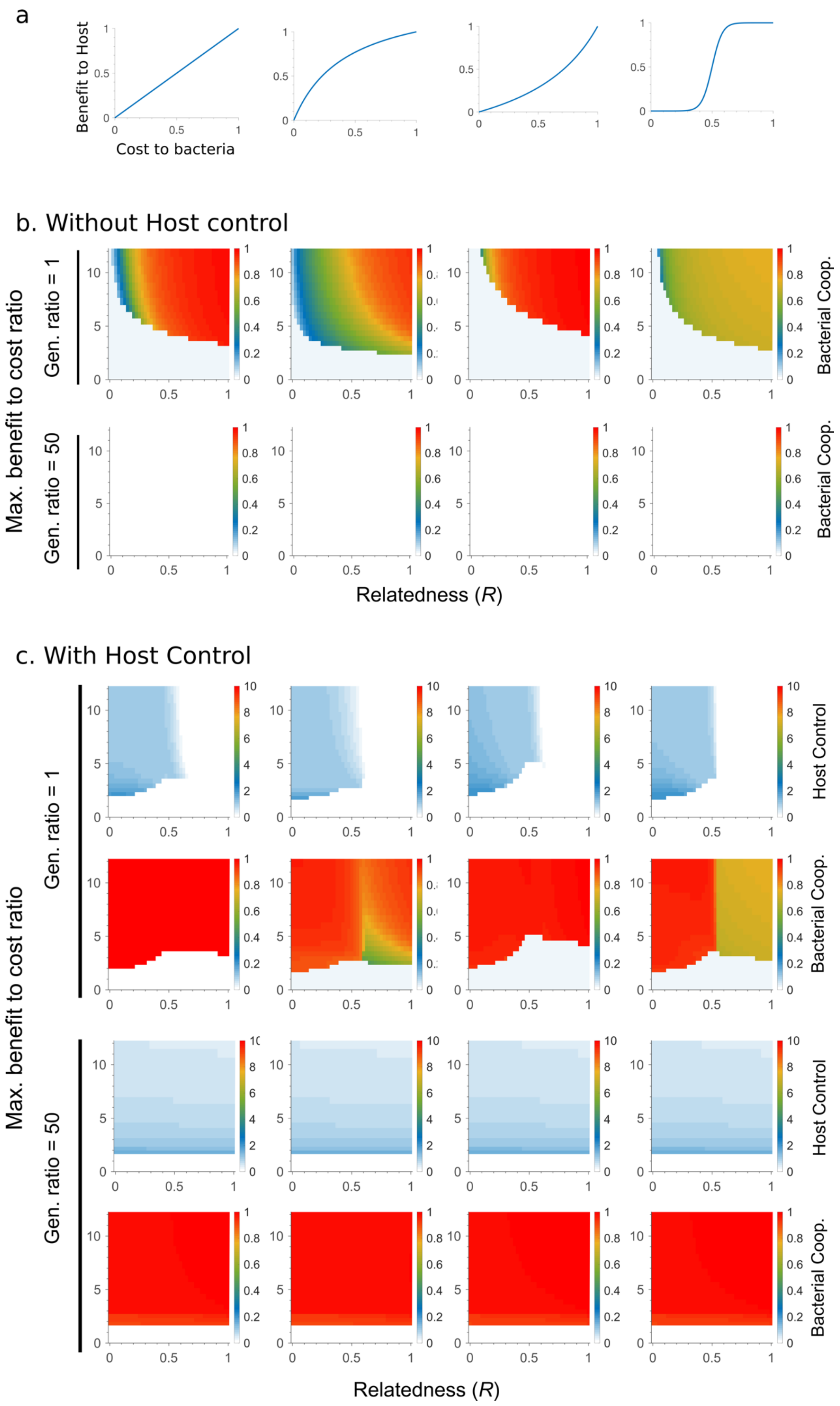

Supplementary Figure 1. The conditions for the evolution of cooperation for different relationships between the investment in cooperation by symbiont and the benefits to a host. Previous work has found that this relationship - between investment in cooperation and the benefit received - can be critical for the evolution of cooperation in some contexts [1], [2]. However, while the functional forms have some effect for low generation ratios, our model predicts that none are able to maintain cooperation at high generation ratios without the addition of host control to our model. a) Functional forms b) Cooperation is widely predicted for a standard linear relationship (as typically used here), a diminishing returns relationship, an accelerating returns relationship, and a sigmoidal relationship. For all relationships, cooperation is lost at higher generation ratios. c) For all relationships host control stabilises cooperation. Unless stated, parameters are $x = y = 2$, $f = 0.02$, $g = 0.1$, $m = 10^{-6}$, $M = 0.05$.

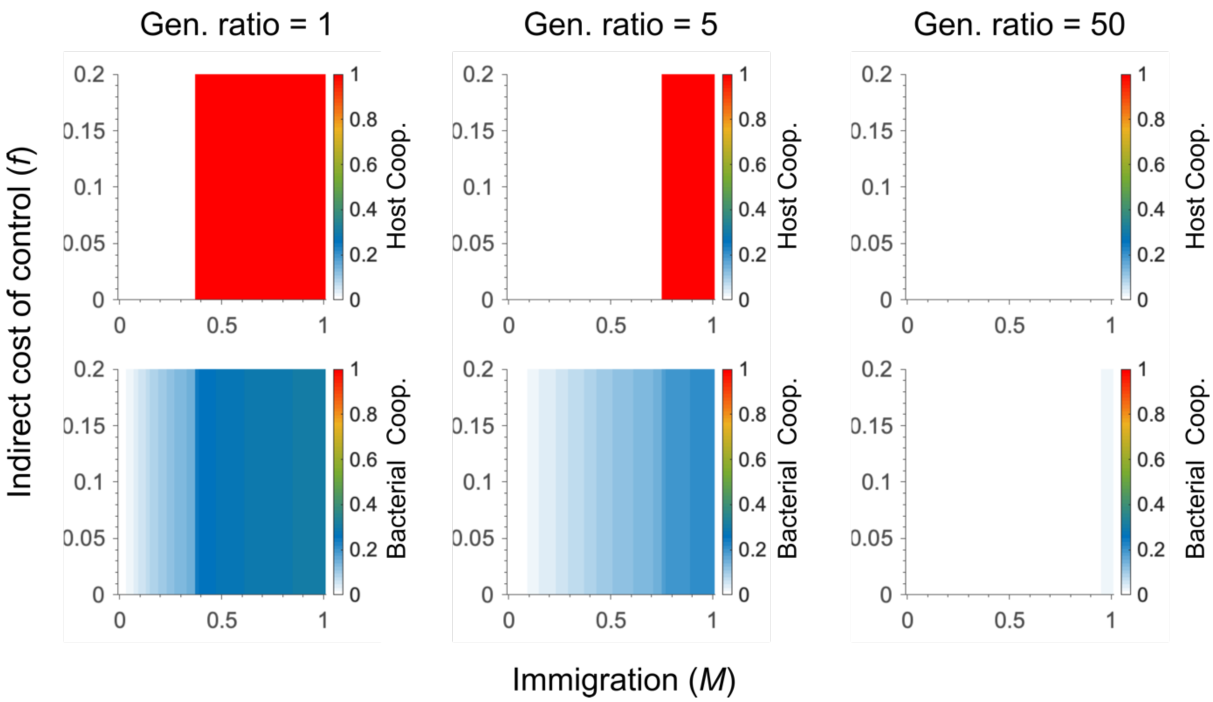

Supplementary Figure 2. The effect of microbial immigration from the environmental pool when there is no host control. High rates of immigration brings in some cooperative genotypes from the environmental pool, which can force cooperation up and generate reciprocal host cooperation. However, as the generation ratio is increased and competition between symbionts within hosts is prolonged, non-cooperative genotypes are selected and cooperation collapses. Unless stated, parameters are $R = 0$, $x = y = 2$, $g = 0.1$, $m = 1\text{x}10^{-6}$.

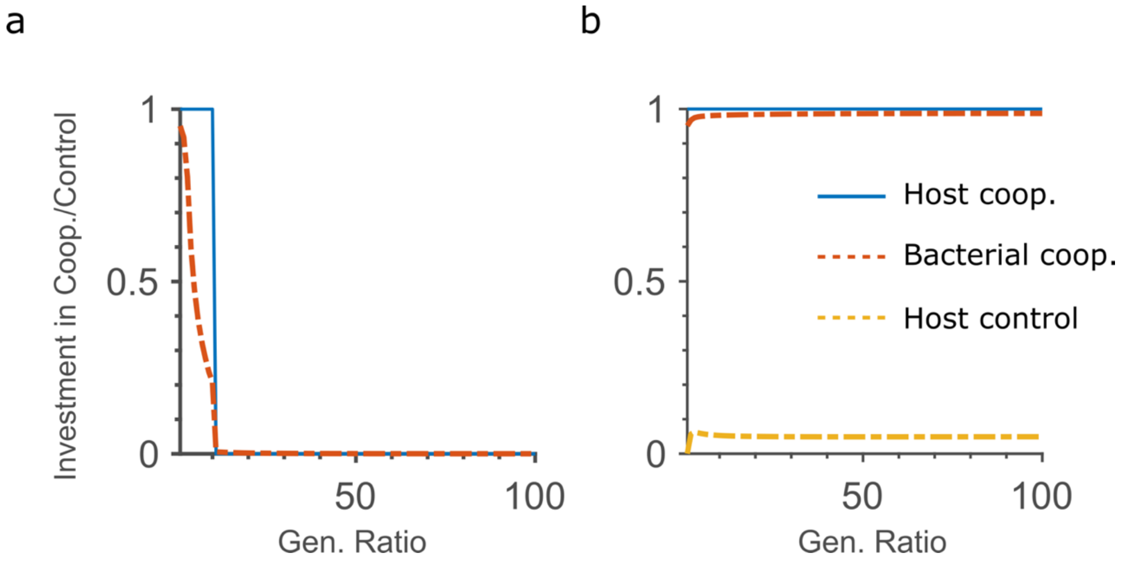

Supplementary Figure 3. Modelling predictions motivated by the human microbiome. Here we parameterise using the estimate of relatedness within symbionts in the human microbiome [3]. Above relatively modest generation ratios, the model predicts that a) cooperation will collapse in microbiomes that lack host control but b) the addition of host control stabilises mutualism. Exact generation ratios in the human microbiome are challenging to estimate but are expected to be much higher than the threshold shown here above which predictions remain unchanged e.g. if bacteria divide every 6 hours, the generation ratio for one year in a human host would be around 1500. Unless stated parameters are $x = y = 3.16$, $R = 0.7$, $f = 0.02$, $g = 0.1$, $m = 1 \times 10^{-6}$, $M = 0.05$.

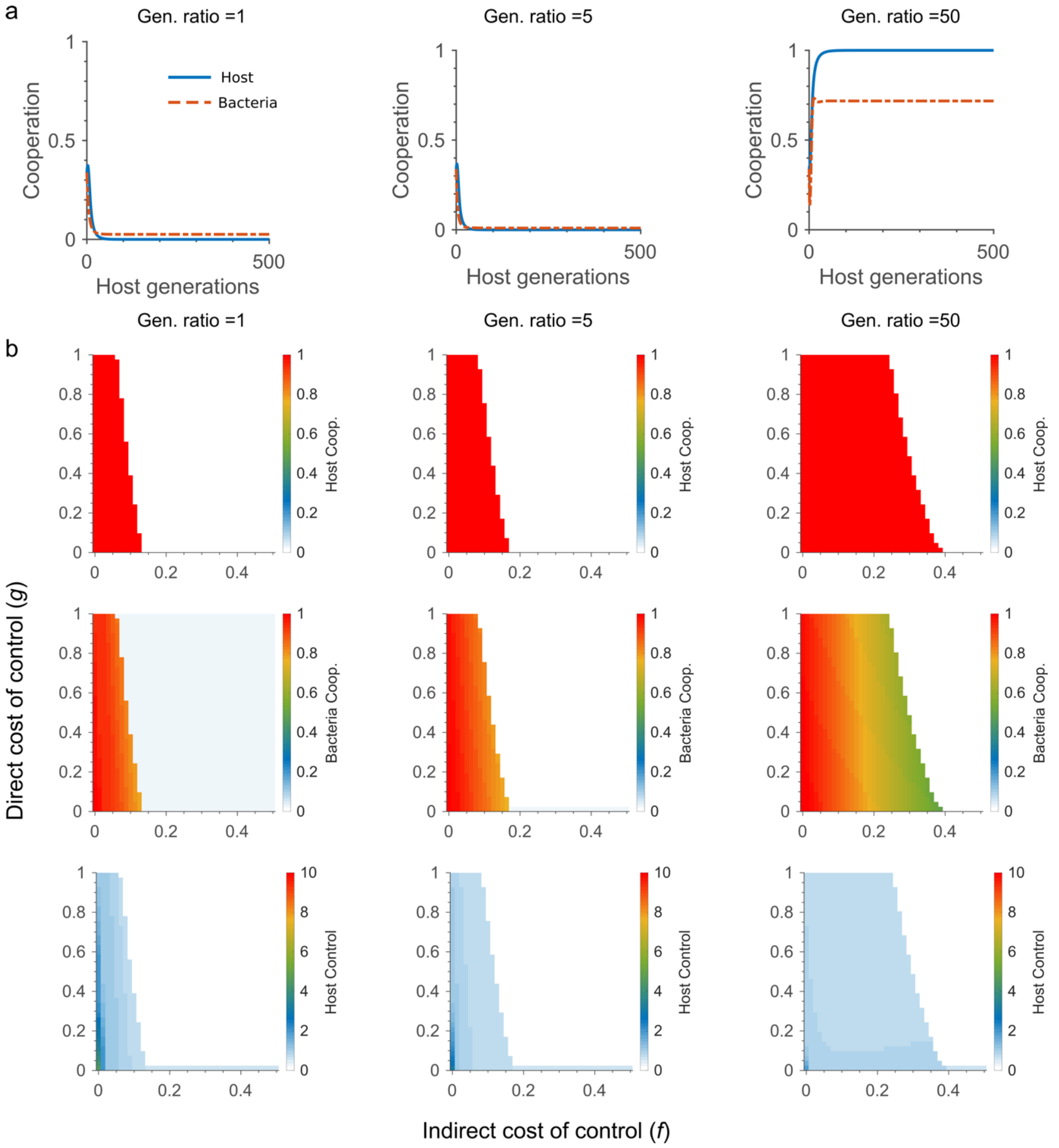

Supplementary Figure 4. The evolution of cooperation under host control as a function of the cost of control on symbionts (*f*) and hosts (*g*). a) Example dynamics where host control is very costly to the host and is only selected at high generation ratios (parameters, unless stated, are $x = y = 2$, $f = 0.2$, $g = 0.4$, $m = 10^{-6}$, $M = 0.05$). b) Parameter sweeps of the direct (*g*) and indirect (*f*) cost of control. Direct here refers to the cost of control to the host e.g. the physiological cost of having an immune system. Indirect refers to the cost of control to the symbionts e.g. how much the population size of symbionts is reduced when the host exercises control, which then feeds back as an indirect cost to the host because it reduces the overall abundance of beneficial symbionts. As for previous analyses, we see here that increasing the microbe to host generation ratio increases the conditions under which control and cooperation will evolve.

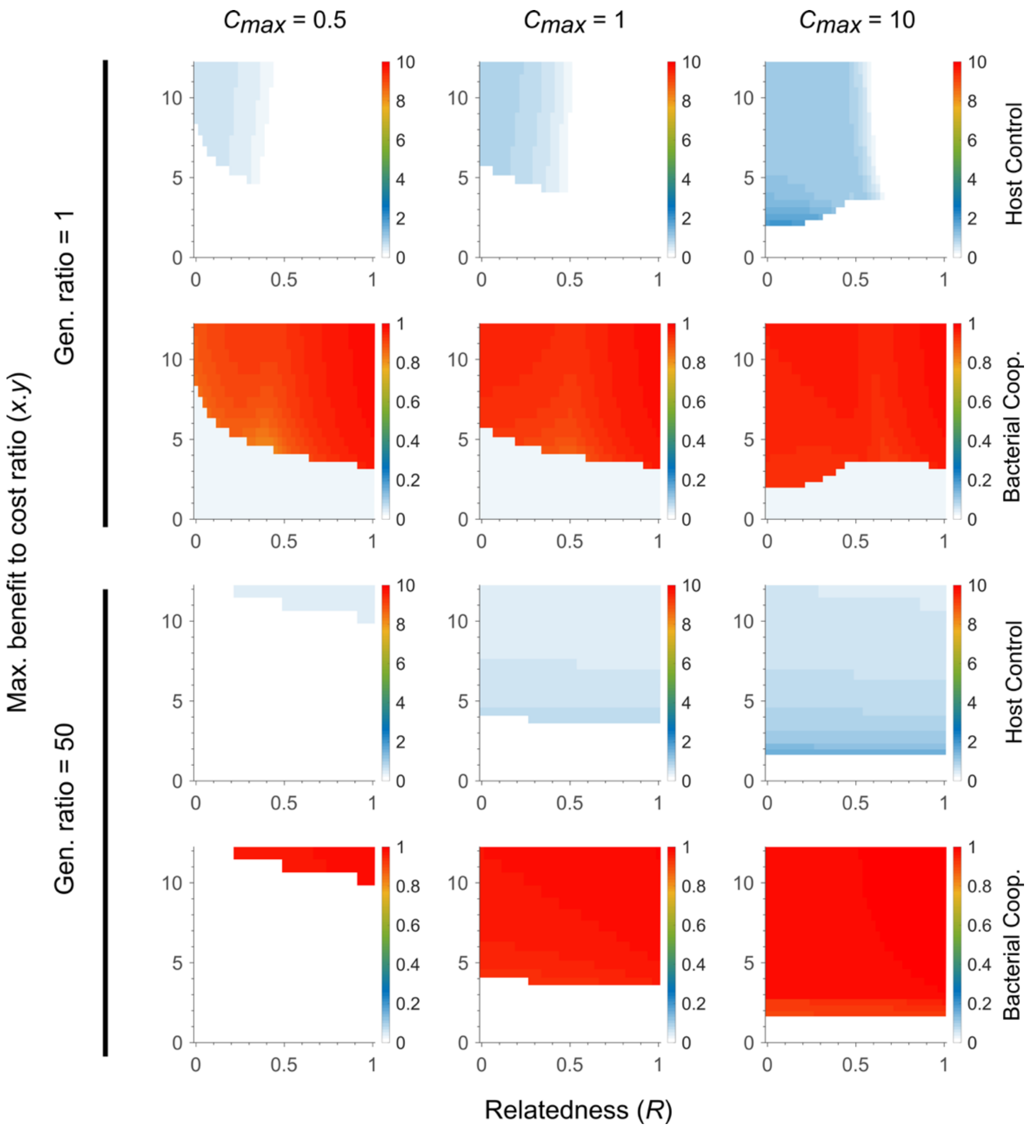

Supplementary Figure 5. Increasing the maximum strength of host control (C_{max}) increases the parameter range where cooperation evolves. Unless stated, parameters are: $f = 0.02$, $g = 0.1$, $m = 1\text{x}10^{-6}$, $M = 0.05$.

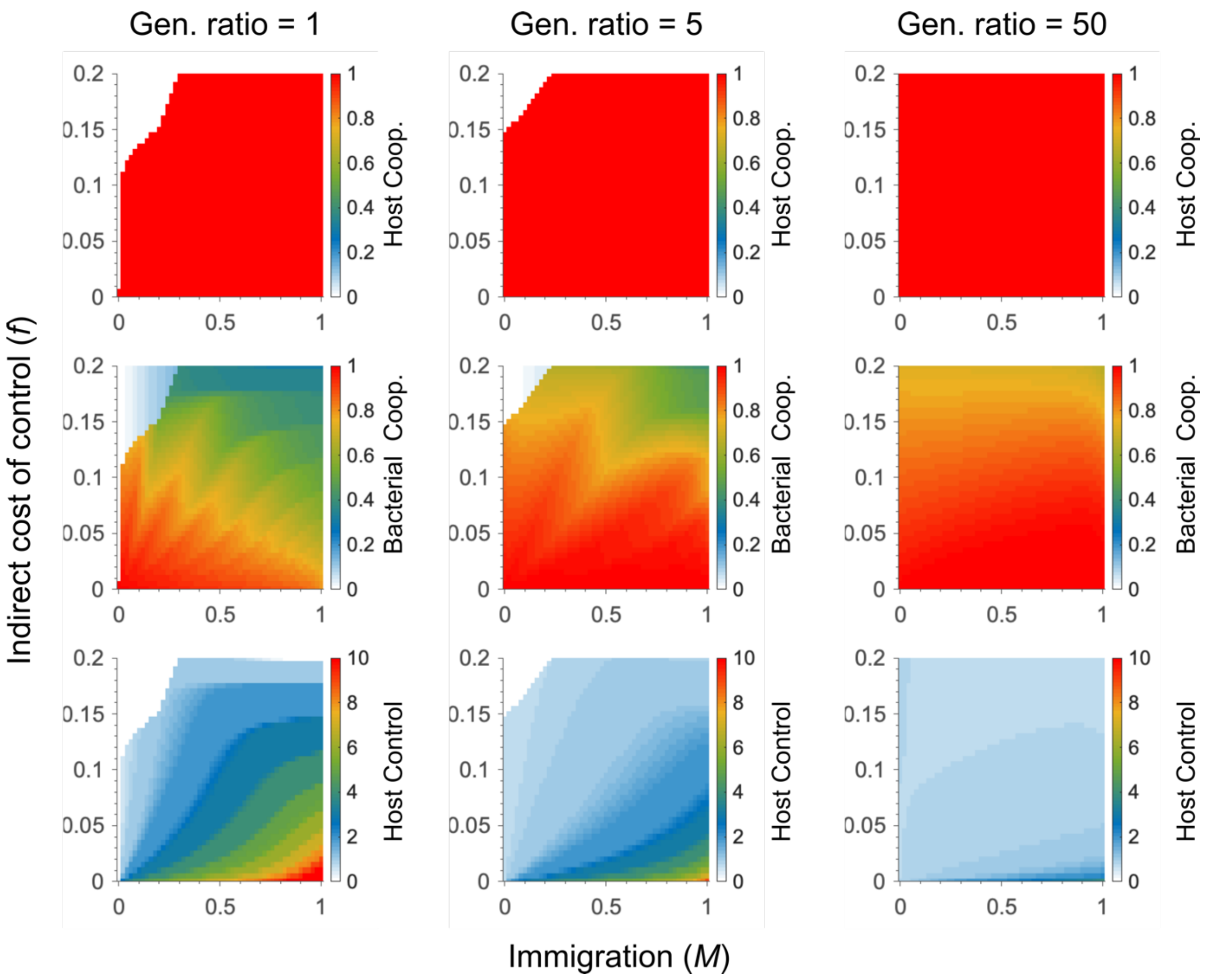

Supplementary Figure 6. Host control evolution is not significantly affected by the immigration of bacteria from the environment into the hosts. Unless stated parameters are $x = y = 2$, $R = 0$, $g = 0.1$, $m = 1 \times 10^{-6}$.

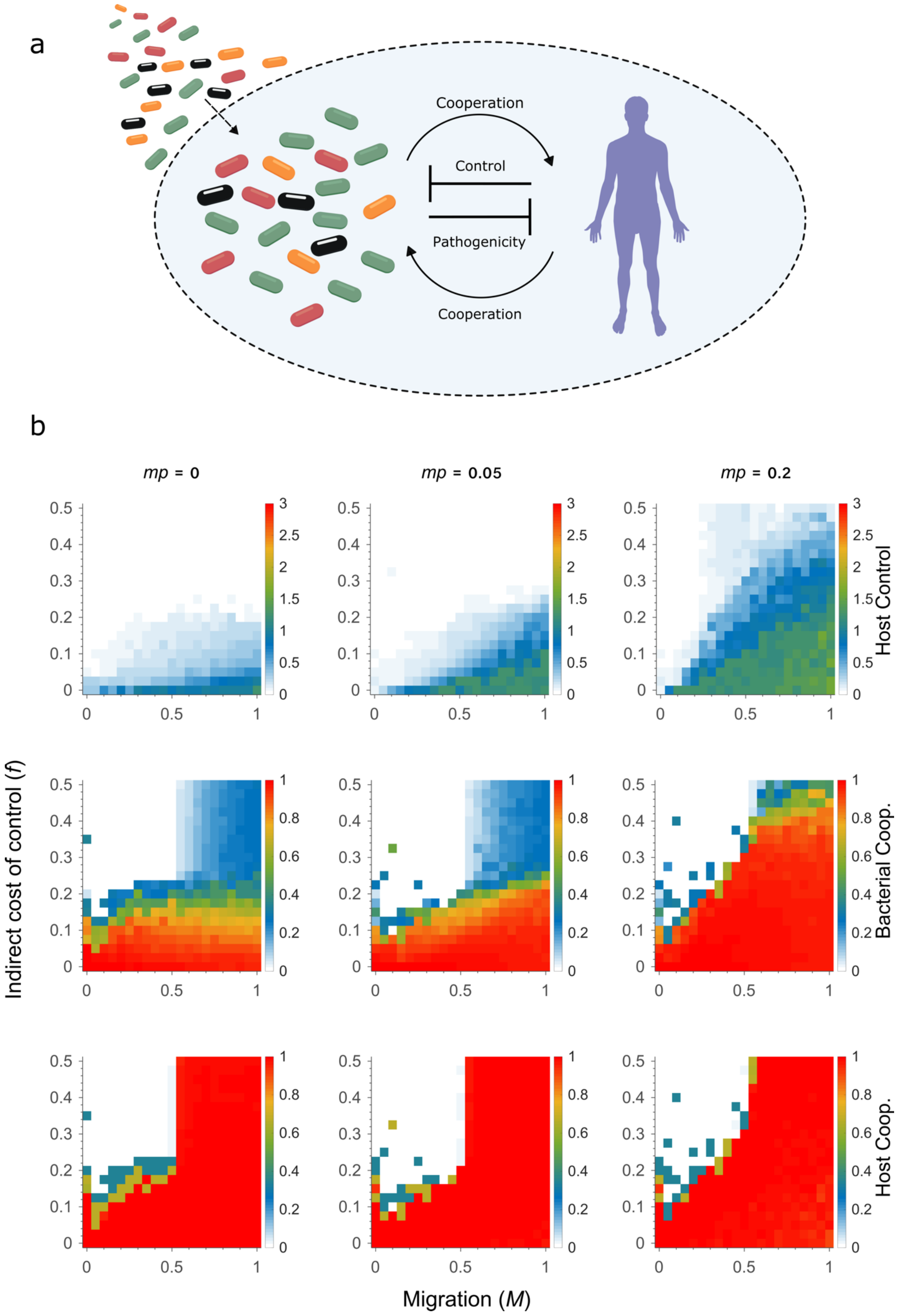
a
Cooperation
Control
Pathogenicity
Cooperation
b
mp = 0
mp = 0.05
mp = 0.2
Host Control
Bacterial Coop.
Host Coop.
Indirect cost of control (f)
Migration (M)

Supplementary Figure 7. Pathogens can promote host control and thereby cooperation with the microbiota. a) Schematic of the model. In order to be able to model the effects of individual pathogen genotypes, we developed an individual based version of the model with 10000 hosts each capable of hosting 1000 microbial symbionts. Pathogens were both non-cooperative and directly harmed the host. b) Increasing the immigration of pathogens increases the range of conditions under which control and cooperation evolve. A proportion p of all immigrating microbes are pathogens. Unless stated, parameters are $x = y = 5$, $g = 0.1$.

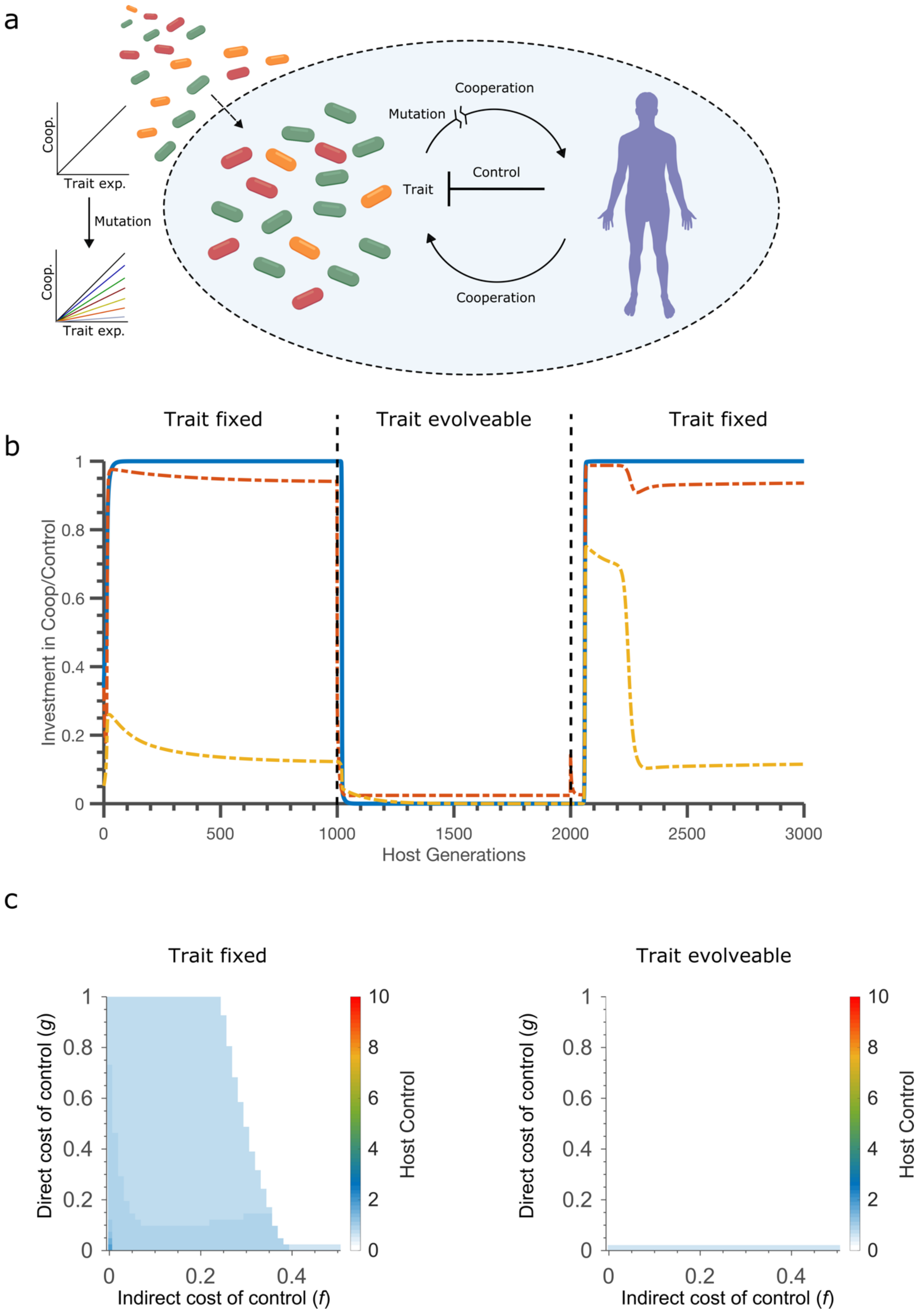

Supplementary Figure 8. Cooperation collapses when symbionts can evolve cooperation independently of the target of host control. a) Schematic of the model. Our original model assumed a fixed linear

relationship between the expression of the bacterial trait that is targeted by host control, and the level of bacterial cooperation. Here we explore what happens when bacteria can evolve to weaken the link between the target of control and cooperation. b) Cooperation between host and symbionts is stable while the trait and cooperation are fixed (original model) but when symbionts are allowed to evolve the trait-cooperation link, cooperation and control are quickly lost. Reinstating the relationship again renders host control effective and restores cooperation. c) Parameter sweeps when the trait-cooperation link is evolvable show cooperation is unstable across a wide range of conditions. Loss of cooperation is independent of the cost of control and generations. When there is no cost of control (g = 0), host control is neutral and remains at level of the initial host population (blue shading at bottom of the plots) Unless otherwise stated parameters are: x = y = 2, $R = 0, f = 0.02, g = 0.1, m = 1x10^{-6}, M = 0.05$.

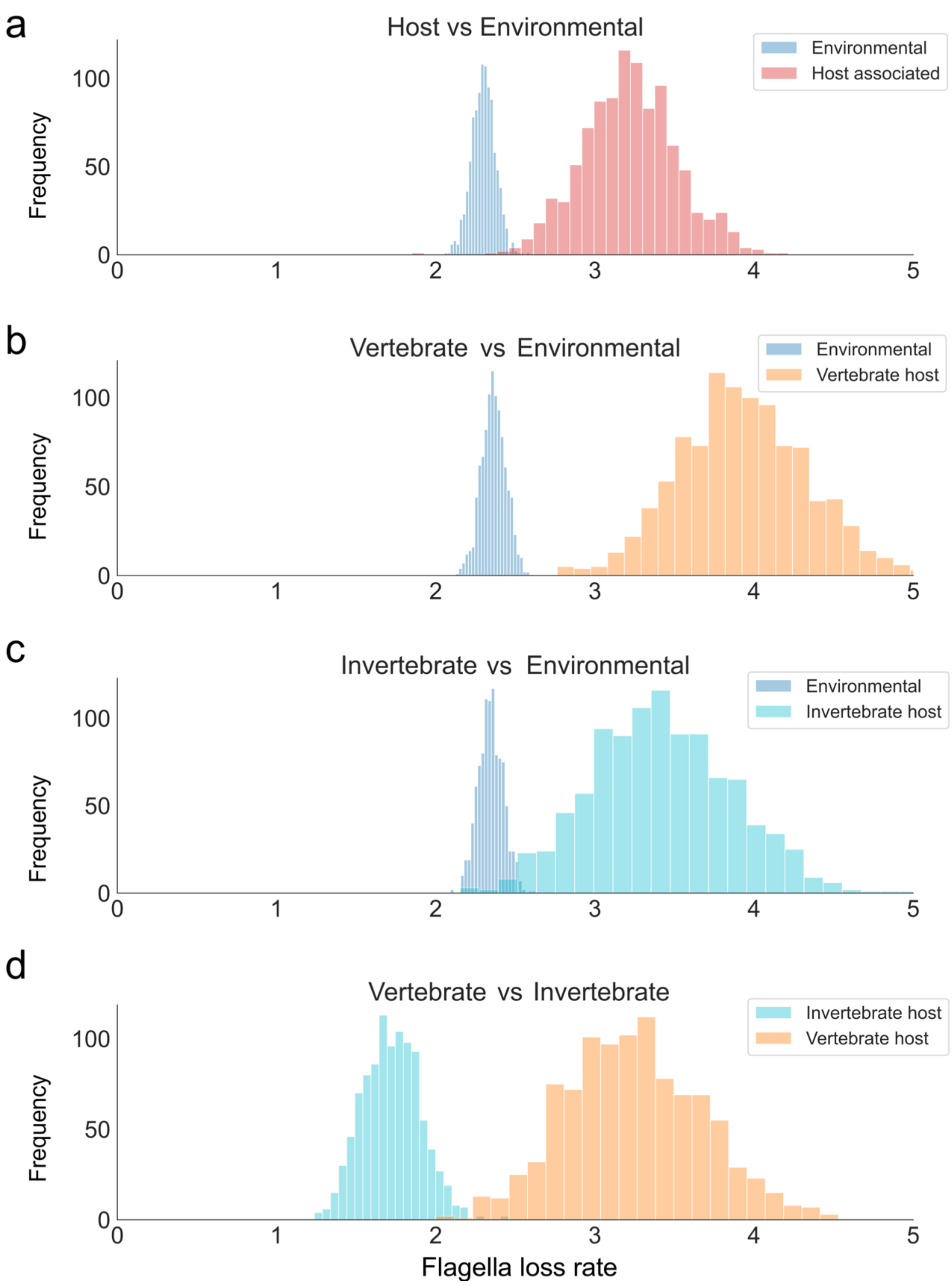

Supplementary Figure 9. Posterior distributions of flagella loss rates in a second data set. Host association and possession of flagella are again negatively correlated, and this data set allowed us to compare a larger number of invertebrate-associated bacteria with vertebrates-associated bacteria. This reveals that flagella loss rate is significantly higher in bacteria associated with vertebrates than those associated with invertebrates (LogBF = 6.14). a) Flagella loss rate in host-associated vs environmental bacteria b) vertebrate host-associated vs environmental c) invertebrate host-associated vs environmental

d) vertebrate vs invertebrate host-associated. Data are from the Genomes of Earth's microbiomes dataset [4]. Source data are provided as a Source Data file.

Supplementary References

[1] K. R. Foster, "Diminishing returns in social evolution: the not-so-tragic commons," *J. Evol. Biol.*, vol. 17, no. 5, pp. 1058–1072, Sep. 2004.

[2] M. Archetti, "Cooperation as a volunteer's dilemma and the strategy of conflict in public goods games," *J. Evol. Biol.*, vol. 22, no. 11, pp. 2192–2200, Nov. 2009.

[3] C. Simonet and L. McNally, "Kin selection explains the evolution of cooperation in the gut microbiota," *Proc. Natl. Acad. Sci.*, vol. 118, no. 6, 2021.

[4] S. Nayfach *et al.*, "A genomic catalog of Earth's microbiomes," *Nat. Biotechnol.*, 2020.
