# Supplementary material for: Host control and the evolution of cooperation in host microbiomes
Source: Nat Commun. 2022 Jun 22;13:3567. doi: 10.1038/s41467-022-30971-8 (PMC9218092; doi:10.1038/s41467-022-30971-8)
Supplement: Supplementary file 2 — Reporting Summary [file 41467_2022_30971_MOESM2_ESM.pdf]

nature portfolio

Corresponding author(s): Prof. Kevin Foster

Last updated by author(s): 22/05/2022

Reporting Summary

Nature Portfolio wishes to improve the reproducibility of the work that we publish. This form provides structure for consistency and transparency in reporting. For further information on Nature Portfolio policies, see our Editorial Policies and the Editorial Policy Checklist.

Statistics

For all statistical analyses, confirm that the following items are present in the figure legend, table legend, main text, or Methods section.

n/a	Confirmed	
☐	☒	The exact sample size (*n*) for each experimental group/condition, given as a discrete number and unit of measurement
☐	☒	A statement on whether measurements were taken from distinct samples or whether the same sample was measured repeatedly
☐	☒	The statistical test(s) used AND whether they are one- or two-sided<br>*Only common tests should be described solely by name; describe more complex techniques in the Methods section.*
☒	☐	A description of all covariates tested
☐	☒	A description of any assumptions or corrections, such as tests of normality and adjustment for multiple comparisons
☒	☐	A full description of the statistical parameters including central tendency (e.g. means) or other basic estimates (e.g. regression coefficient) AND variation (e.g. standard deviation) or associated estimates of uncertainty (e.g. confidence intervals)
☐	☒	For null hypothesis testing, the test statistic (e.g. *F*, *t*, *r*) with confidence intervals, effect sizes, degrees of freedom and *P* value noted<br>*Give P values as exact values whenever suitable.*
☐	☒	For Bayesian analysis, information on the choice of priors and Markov chain Monte Carlo settings
☐	☒	For hierarchical and complex designs, identification of the appropriate level for tests and full reporting of outcomes
☒	☐	Estimates of effect sizes (e.g. Cohen's *d*, Pearson's *r*), indicating how they were calculated

Our web collection on statistics for biologists contains articles on many of the points above.

Software and code

Policy information about availability of computer code

Data collection	No software was used for data collection
Data analysis	Evolutionary Modeling was performed and analysed using Matlab R2018a using scripts found at https://github.com/Connor-Sharp/CoevolutionModel. Baysian analysis was performed using BayestraitsV3. To generate phylogenies, sequences were aligned using Clustal Omega v1.2.4 and phylogeny calculated using FastTreeV2. The R packages Phylotools and Simmap were used to estimate the number of state transitions on the tree. Phylogenies were visualized using iTOL. Histograms of bayestraits transition rates were generated using Python and the package seaborn.

For manuscripts utilizing custom algorithms or software that are central to the research but not yet described in published literature, software must be made available to editors and reviewers. We strongly encourage code deposition in a community repository (e.g. GitHub). See the Nature Portfolio guidelines for submitting code & software for further information.

Data

Policy information about availability of data

All manuscripts must include a data availability statement. This statement should provide the following information, where applicable:

- Accession codes, unique identifiers, or web links for publicly available datasets
- A description of any restrictions on data availability
- For clinical datasets or third party data, please ensure that the statement adheres to our policy

Genomic data used in this study was accessed from publicly available datasets: Pathosystems Resource Integration Center (PATRIC) reference genomes (https://

www.patricbrc.org) and Genomes of earths microbiomes (available at https://img.jgi.doe.gov/ and https://portal.nersc.gov/GEM). Additional metadata was accessed from the publicly available dataset BacDive (https://bacdive.dsmz.de). Hidden markov models used in identifying flagellin and butyrate related genes were accessed from PFAM (https://pfam.xfam.org) and all accessions are listed in Table 2.

Human research participants

Policy information about studies involving human research participants and Sex and Gender in Research.

Reporting on sex and gender	*Use the terms sex (biological attribute) and gender (shaped by social and cultural circumstances) carefully in order to avoid confusing both terms. Indicate if findings apply to only one sex or gender; describe whether sex and gender were considered in study design whether sex and/or gender was determined based on self-reporting or assigned and methods used. Provide in the source data disaggregated sex and gender data where this information has been collected, and consent has been obtained for sharing of individual-level data; provide overall numbers in this Reporting Summary. Please state if this information has not been collected. Report sex- and gender-based analyses where performed, justify reasons for lack of sex- and gender-based analysis.*
Population characteristics	*Describe the covariate-relevant population characteristics of the human research participants (e.g. age, genotypic information, past and current diagnosis and treatment categories). If you filled out the behavioural & social sciences study design questions and have nothing to add here, write "See above."*
Recruitment	*Describe how participants were recruited. Outline any potential self-selection bias or other biases that may be present and how these are likely to impact results.*
Ethics oversight	*Identify the organization(s) that approved the study protocol.*

Note that full information on the approval of the study protocol must also be provided in the manuscript.

Field-specific reporting

Please select the one below that is the best fit for your research. If you are not sure, read the appropriate sections before making your selection.

☒ Life sciences ☐ Behavioural & social sciences ☐ Ecological, evolutionary & environmental sciences

For a reference copy of the document with all sections, see nature.com/documents/nr-reporting-summary-flat.pdf

Life sciences study design

All studies must disclose on these points even when the disclosure is negative.

Sample size	Sample sizes were limited by the sizes of the publicly available datasets: Pathosystems Resource Integration Center (PATRIC) reference genomes (https://www.patricbrc.org) and Genomes of earths microbiomes (available at https://img.jgi.doe.gov/ and https://portal.nersc.gov/GEM).
Data exclusions	To maximize phylogenetic coverage and prevent sampling bias from overrepresented taxa (e.g. E. coli), PATRIC genomes were limited to the representative dataset, a pre-defined dataset available on the PATRIC website (https://www.patricbrc.org).
Replication	Individual based models as used to generate Supplementary Figure 7 were repeated in triplicate with different random seeds and averaged over the three replicates.
Randomization	As whole datasets were used randomization was not possible. Analysis was performed to ensure that our results were not dependent on only a small number of transitions and BayesTraits analysis was performed across 10 calculated trees and tested against random label switching to ensure phylogenetic bias was not dictating the observed trends.
Blinding	This study was not amenable to blinding. Data collection and analysis was performed by a single author rendering blinding impossible.

Reporting for specific materials, systems and methods

We require information from authors about some types of materials, experimental systems and methods used in many studies. Here, indicate whether each material, system or method listed is relevant to your study. If you are not sure if a list item applies to your research, read the appropriate section before selecting a response.

Materials & experimental systems

n/a	Involved in the study
☒	☐ Antibodies
☒	☐ Eukaryotic cell lines
☒	☐ Palaeontology and archaeology
☒	☐ Animals and other organisms
☒	☐ Clinical data
☒	☐ Dual use research of concern

Methods

n/a	Involved in the study
☒	☐ ChIP-seq
☒	☐ Flow cytometry
☒	☐ MRI-based neuroimaging
